# Intermediate stages in the origin of metabolism at a phosphorylating hydrothermal vent

**DOI:** 10.1126/sciadv.aef3128

**Published:** 2026-08-05

**Authors:** Natalia Mrnjavac, Nadja K. Hoffmann, Manon L. Schlikker, Maximilian Burmeister, Loraine Schwander, Carolina García García, Max Brabender, Mike Steel, Daniel H. Huson, Sabine Metzger, Quentin Dherbassy, Bernhard Schink, Mirko Basen, Joseph Moran, Harun Tüysüz, Martina Preiner, William F. Martin

**Affiliations:** ^1^ Institute of Molecular Evolution, Faculty of Mathematics and Natural Sciences, Heinrich Heine University Düsseldorf, Düsseldorf, Germany.; ^2^ Biomathematics Research Centre, University of Canterbury, Christchurch, New Zealand.; ^3^ Institute for Bioinformatics and Medical Informatics, University of Tübingen, Tübingen, Germany.; ^4^ Biocenter MS-Platform, University of Cologne, Cologne, Germany.; ^5^ University of Strasbourg, CNRS, ISIS, Strasbourg, France.; ^6^ Department of Biology, University of Konstanz, Konstanz, Germany.; ^7^ Department of Microbiology, Institute of Biological Sciences, University of Rostock, Rostock, Germany.; ^8^ Department of Chemistry and Biomolecular Sciences, University of Ottawa, Ottawa, Canada.; ^9^ Catalysis and Energy Materials Group, IMDEA Materials Institute, Madrid, Spain.; ^10^ Department of Heterogeneous Catalysis, Max-Planck-Institut für Kohlenforschung, Mülheim an der Ruhr, Germany.; ^11^ Microcosm Earth Center, Max Planck Institute for Terrestrial Microbiology and Philipps University Marburg, Marburg, Germany.

## Abstract

The origin of life required the emergence of metabolism, an autocatalytic network of enzymatic reactions that synthesize amino acids, nucleotides, and cofactors. At the origin of metabolism, there were no enzymes—how did it start? Empirical studies addressing early metabolic evolution are lacking. Harnessing protein structures for metabolic enzymes, we identify intermediate states in primordial metabolic assembly. We show that enzymatic metabolism in the universal common ancestor was incomplete, undergoing final assembly independently in the lineages leading to bacteria and archaea. Native transition metals—iron, cobalt, nickel, and palladium—served as the catalytic forerunners of both enzymes and cofactors at metabolic origin, while phosphite supplied energy, as it phosphorylates adenosine 5′-monophosphate to adenosine 5′-diphosphate and serine to phosphoserine using native metal catalysts in water. Phosphite and native metals occur in serpentinizing hydrothermal systems, identifying an energy-supplying, catalytic site of metabolic origin. Cofactors liberated nascent metabolism from native metal catalysts, engendering its autocatalytic state.

## Teaser

Enzymatic metabolism emerged from metal-catalyzed, aqueous reactions of H_2_, CO_2_, NH_3_ and phosphite at a hydrothermal vent.

## INTRODUCTION

Metabolism is a non-negotiable property of life. It transforms environmental compounds into the building blocks of life and the chemical substance of cells. Although modern metabolism is catalyzed by enzymes, at origins, there were no enzymes, leaving only the environment as the source of compounds and catalysts that spawned the first metabolic networks. However, the environment in which life and metabolism arose is hotly debated, suggestions including ultraviolet (UV)-rich terrestrial hot springs (*1*), terrestrial cyanide deposits (*2*), volcanos (*3*, *4*), ice (*5*), and submarine hydrothermal vents (*6*–*8*). Because the environment of origins conditions its chemistry, almost everything about the origin of metabolism is debated, including the roles of energy (*9*), genetics (*10*), autocatalysis (*11*), phosphate (*12*, *13*), cofactors (*14*), cyanide (*15*), CO_2_ (*3*), and water (*16*). However, on one aspect, all will agree: The ∼400-reaction network that converts H_2_, CO_2_, NH_3_, H_2_S, and phosphate into amino acids, nucleobases, and cofactors (*17*) cannot have arisen in an instant. Its emergence from spontaneous environmental reactions had to traverse intermediate states of assembly, which have previously been elusive.

A key question about metabolic origin concerns its state in the last universal common ancestor (LUCA) (*18*, *19*). Was LUCA able to synthesize all of its metabolic intermediates itself or were some still supplied by the environment, and were all of LUCA’s reactions catalyzed by enzymes or did the environment supply catalysts that served as the forerunners of LUCA’s enzymes? In addition, if the environment supplied compounds and catalysts for LUCA, then how far did that dependency extend into the lineages leading to the last common ancestors of bacteria and archaea (LBCA and LACA), respectively? The processes of enzyme origins and metabolic assembly across the deepest divide of the tree of life have hitherto not been studied.

The 400 reactions of metabolism and corresponding enzymes can provide insights into the earliest phases of biological divergence, but they might also harbor evidence for the kind of environment within which metabolism arose. Do reactions and enzymes that trace to LUCA indicate a different kind of chemistry or a different environment from those that trace to LACA and LBCA, and can the different theories for the site of life’s origin be discriminated on the basis of metabolism’s chemistry? Neither question has been explored on the basis of metabolism’s conserved 400 reaction set.

Perhaps the steepest barrier to understanding metabolic origin concerns the role of energy and phosphate. Regardless of how or where metabolism arose, its reactions must have been thermodynamically favorable; otherwise, they would not have gone forward, and life’s metabolic network never would have arisen. Stated another way, in thermodynamically favorable reactions, the equilibrium lies on the side of products rather than reactants. Some reactions in metabolism are thermodynamically unfavorable, but can nonetheless go forward if subsequent reactions remove products (*20*) (sometimes called pulling reactions in metabolism) or if the reaction is coupled to a highly exergonic reaction such as adenosine 5′-triphosphate (ATP) hydrolysis (*21*). Thus, as with modern metabolism, the overall energetics of primordial metabolism had to be favorable in order for it to go forward. Central to energetics in metabolism is phosphate (*22*). Phosphate is poorly soluble and does not readily form reactive anhydrides or organophosphate compounds in water (*22*), prompting proposals that metabolism either arose in environments that underwent recurrent desiccation, which promotes phosphorylation reactions (*22*, *23*) but denatures enzymes, or arose without phosphorus altogether (*24*). The latter suggestion seems unlikely, as the conserved reactions of amino acid, nucleotide, and cofactor synthesis involve 360 intermediates, 114 of which contain one or more phosphate residues—phosphate has been part of metabolism since there were genes. Compounds capable of phosphorylation had to exist in the environment where metabolism arose, but environmental sources of aqueous phosphorylation have not been identified to date.

To chart the course of early metabolic evolution, we harnessed information in protein structures for metabolic enzymes to identify the enzymes present in the metabolism of LUCA and the growth of that reaction network in the independent lineages leading to bacteria and archaea. This identified intermediate states in primordial metabolic assembly in which enzymatic metabolism was incomplete, with missing enzymes and intermediates being supplied by native transition metals—Fe^0^, Co^0^, Ni^0^, and their alloys—which are known to catalyze essential metabolic pathways (*25*–*30*) and which are naturally deposited in serpentinizing hydrothermal systems (*31*–*33*). Guided by microbes that use phosphite as their sole source of phosphorus (*34*, *35*), we found that another native metal of serpentinizing hydrothermal vents (*32*, *33*), Pd^0^, provided a hitherto missing source of phosphorylation at origins, as it catalyzed phosphite-dependent phosphorylation of adenosine 5′-monophosphate (AMP) and serine in water. Phosphite also occurs in serpentinizing systems (*12*), its activation to phosphate in water is highly exergonic (*22*), identifying a natural supply of continuous chemical energy and implicating serpentinizing hydrothermal vents as a thermodynamically favorable site of metabolic assembly.

## RESULTS AND DISCUSSION

### Core metabolism in LUCA was incomplete

To identify intermediate states in the primordial assembly of metabolism, we harnessed information available in enzyme sequences and structures, which can detect more ancient homologies among proteins than sequence comparisons alone (*36*). Starting from a balanced set of genomes encompassing 552 bacterial and 401 archaeal isolates with representation in all major groups and excluding metagenomic assembled genomes, we generated structure-refined clusters of enzymes that map to 361 reactions required to synthesize amino acids, nucleobases, and cofactors from gasses (H_2_, CO_2_, NH_3_, and H_2_S) and mineral salts (see Materials and Methods). The standard Markov Cluster Algorithm (MCL) sequence clustering procedure initially generated 1473 clusters, which were merged by structural similarity, yielding 426 structurally refined clusters (fig. S1).

**Fig. 1.**
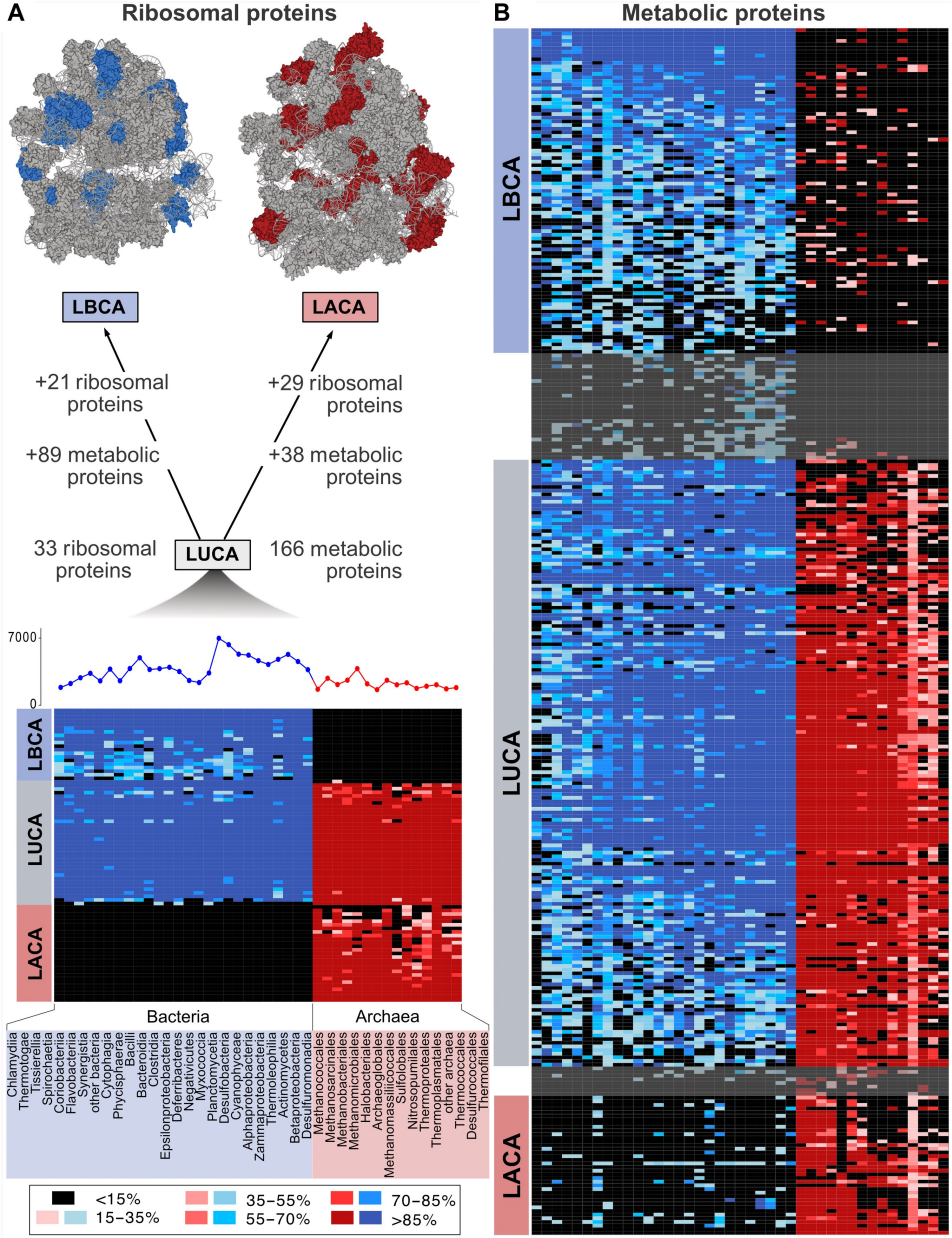
Distribution of ribosomal and core metabolism protein families across bacterial classes and archaeal orders. Protein families in bacterial genomes are plotted as blue ticks, and archaeal are plotted as red ticks. Color intensity indicates the proportion of genomes per taxon possessing the gene (scale at lower left). (**A**) Universal ribosomal proteins (tracing to LUCA) are gray in the ribosome structures, bacterial-specific ribosomal proteins are shown in blue, and archaeal-specific ribosomal proteins are shown in red. The schematic tree shows the origin of ribosomal proteins (*41*, *42*) and metabolic proteins (this paper). The line graph shows the average genome size (proteins) per taxon. The distribution of LUCA-, bacteria-, and archaea-specific ribosomal proteins displays a characteristic pattern. Taxa are indicated in the bottom panel. (**B**) The distribution of core metabolic protein families mirrors ribosomal protein origin in (A). Protein families attributable to LUCA, LBCA (top), and LACA (bottom) (see Materials and Methods) are marked. Gray zones designate protein families that are sparsely distributed and hence difficult to assign to LUCA or lineages (see Materials and Methods). The probability of observing, by chance, the dichotomy of columns along the bacterial versus archaeal split in (B) is 0.002 (two-sided permutation test).

The diversity of theories for metabolic origin (*3*, *9*–*11*, *15*, *25*, *37*–*40*) generates few predictions for the expected evolutionary trajectory of enzymatic metabolism. The ribosome (*41*, *42*) can, however, serve as a predictive model for the emergence of complex traits. The ribosome of LUCA (*41*, *42*) was simpler than its modern forms (Fig. 1A), containing only 33 ribosomal proteins. It underwent independent lineage-specific assembly via addition of 29 and 21 proteins in lineages leading to LACA and LBCA, respectively (Fig. 1A and table S1) (*41*, *42*). We asked whether the assembly of metabolism, which supplies the RNA and amino acid monomers required for ribosome synthesis and function, proceeded via a ribosome-like trajectory from a simple intermediate state in LUCA, followed by lineage-specific addition of novel reactions.

**Fig. 2.**
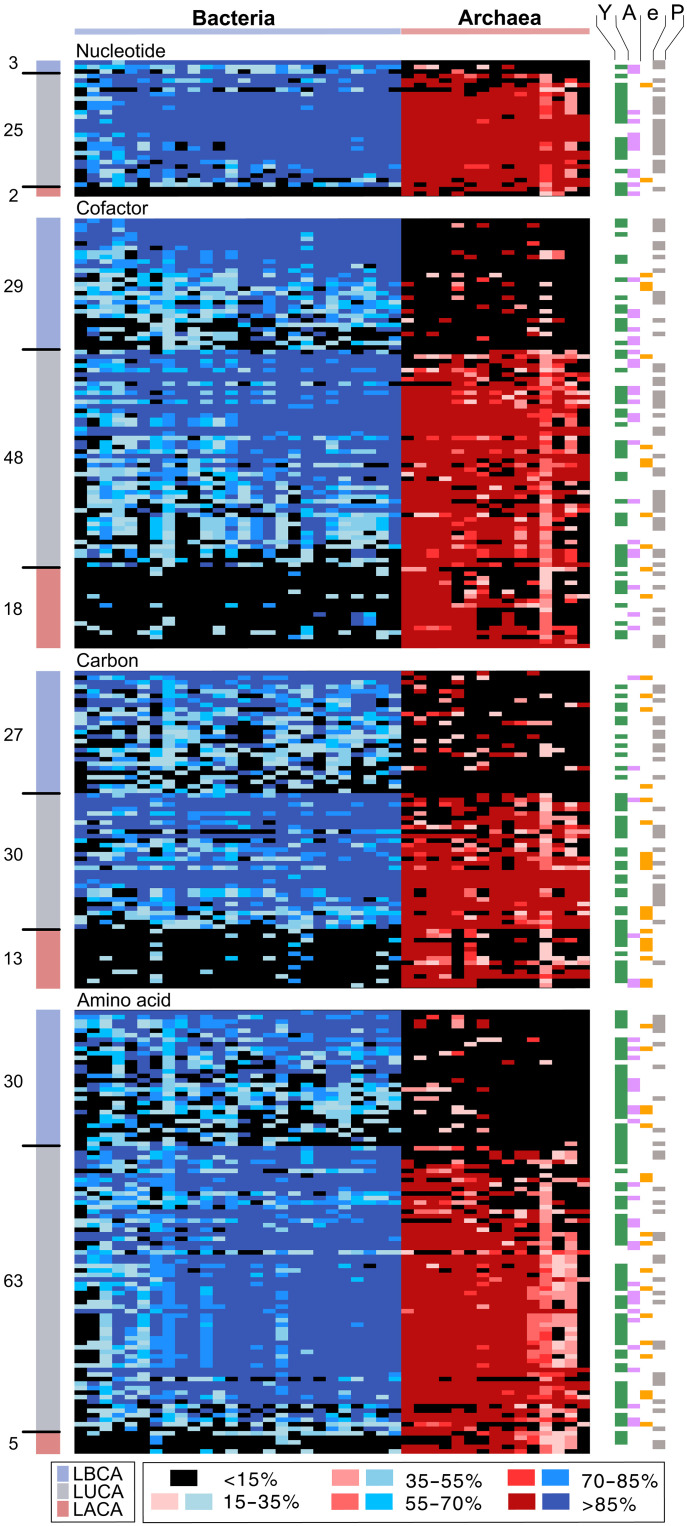
Distribution of core metabolism protein families by sectors of metabolism. The rows are identical to those in Fig. 1B but grouped by function. Gray areas from Fig. 1B are excluded. The number of protein families per category is shown on the left. Columns on the right denote reactions that involve carbonyl groups (Y; green), C─N bond formation (A; pink), electron transfer (e; yellow), and formation or cleavage of phosphate bonds (P; gray), as listed in table S3.

Plotting the phylogenetic distributions of core biosynthetic enzymes across bacteria and archaea reveals that enzymatic metabolism in LUCA was incomplete (Fig. 1B and table S2). It expanded via origins of novel enzymes in the lineages leading to LACA and LBCA, closely mirroring lineage-specific assembly of the ribosome (*41*, *42*). In the present sample, 166 enzymes of core metabolism trace to LUCA, yet 89 enzymes required for the synthesis of amino acids, cofactors, and nucleobases arose on the lineage to LBCA, while 38 arose on the lineage to LACA. An additional 37 enzymes were too sparsely distributed for unequivocal lineage attribution (gray shading in Fig. 1B). These post-LUCA bacterial and archaeal enzyme innovations reveal that the insufficiency of enzymatic metabolism in LUCA was severe and provide insights into an extremely early phase of biochemical evolution. The metabolic reactions of LUCA (Fig. 1B) capture a time in which the ribosome, the genetic code, and translation were functional because enzymes existed and were arising de novo. However, enzymes were evolving without the support of a complete enzymatic supply of precursors for protein synthesis: Amino acid synthesis, cofactor synthesis, and intermediate carbon metabolism were incomplete in LUCA (Fig. 2). How could an incomplete enzymatic metabolism support protein evolution at the ribosome?

The principle of autocatalysis offers one avenue of explanation in that catalysts can generate products that generate new catalysts such that metabolic networks can grow (*11*). However, the problem at the origin of metabolism is not whether autocatalytic networks can arise in nature—they can (*43*)—the problem is practical: Where do sufficient concentrations of the very first amino acids and nucleobases come from that permit the synthesis of ribosomes for the synthesis of metabolic enzymes that set the reactions of life moving forward? Two broad schools of thought exist on the primordial supply of monomers at metabolic origins.

One proposal is that a primordial, cyanide-dependent chemistry disjunct from modern, CO_2_-dependent metabolism generated the first building blocks of life (*15*, *44*). A vast number of prebiotic reactions have been reported that synthesize essential biomolecules yet use reactants that do not occur in metabolism (*15*, *44*, *45*). These traditioned laboratory syntheses demonstrate that biological compounds can arise by varied and elegant abiotic routes, in addition to biosynthetic routes, but they do not speak to the origin or evolution of enzymatic reactions of metabolism as they occur in cells (Fig. 1B and table S2), which, at the level of ecosystems, always start from CO_2_ (*46*). Cyanide-dependent synthesis thus requires two origins of metabolism, one from cyanide and the modern version from CO_2_. An alternative to this two-origin proposal is that naturally existing catalysts, if provided with a continuous environmental source of simple biochemical reactants (H_2_, CO_2_, and NH_3_), promoted CO_2_-based reactions virtually identical to those of modern metabolism (*25*, *30*, *39*, *40*, *47*) and that catalytic activities present in the environment where metabolism arose were later replaced by enzymes and cofactors (*37*–*40*, *48*), allowing the process of metabolic evolution to go forward, both in LUCA and its descendant lineages. This would require only one origin of metabolism, with a continuity of chemical reactions from environment to cells, accompanied, however, by an increasingly complex set of catalysts (enzymes and cofactors), synthesized by metabolism with the effect of reinforcing and accelerating network formation (*43*). To distinguish between these possibilities, we investigated the reactions of metabolism.

### Metabolism harbors evidence for the site of its origin

In both Figs. 1B and 2, the metabolic reactions enzymatically catalyzed in LUCA uncover an intermediate state during metabolic origin that underwent expansion in its descendant lineages. From this intermediate state, a salient observation emerges: While missing enzymes for reactions in LUCA’s metabolism were still evolving, the initial network remained intact in the lineages leading to both LACA and LBCA, as did the initial inputs to the network: H_2_, CO_2_, NH_3_, H_2_S, H_2_O, and inorganic phosphate (P_i_). This unconditionally requires continuity (*40*) in the process of metabolic evolution and its environment, bearing out, a century after its formulation, Kluyver and Donker’s (*49*) central postulate that there is unity in biochemistry, also during its origin. The growth of metabolism reveals continuity during the course of metabolic assembly from LUCA to LACA and LBCA. In much the same way as the genetic code and the ribosome provide strong evidence for a single origin of life (*41*, *42*), the metabolic reactions of LUCA reveal an intermediate state in metabolic evolution (Fig. 1B) that provides strong evidence for a single, continuously reactive chemical environment as the site of metabolic origin.

However, what kind of chemical environment? Again, the reactions of metabolism speak to this question because they identify a starting point: CO_2_ reduction to pyruvate via the acetyl-coenzyme A (CoA) pathway. It is a linear pathway of H_2_-dependent CO_2_ reduction (*46*) and is the only one that operates in both bacteria and archaea (*46*, *47*). It uses Fe, Ni, and Co at the active site of its enzymes for H_2_ activation and CO_2_ conversion to acetyl-CoA (*50*–*52*). It energetically couples H_2_-dependent CO_2_ fixation to ATP synthesis in acetogens (bacteria) and methanogens (archaea) (*53*, *54*). In phylogenetic reconstructions, it traces to LUCA (*18*, *19*), albeit requiring different cofactors (*55*) and unrelated enzymes (*56*) in the archaeal and bacterial methyl synthesis branch. Moreover, under conditions of modern serpentinizing hydrothermal systems (alkaline aqueous solution, H_2_ partial pressures on the order of 5 atm), catalysts that are naturally deposited in serpentinizing systems—Ni^0^, Fe^0^, Ni_3_Fe (*31*), and Fe_3_O_4_ (*57*)—convert H_2_ and CO_2_ in the laboratory overnight at 20° to 100°C in water to formate, acetate, and pyruvate (plus methane), the intermediates and end products of the acetyl-CoA pathway (*25*, *27*, *30*). Serpentinizing hydrothermal vents still generate H_2_ (*58*), native metals (*31*), abiotic formate, and methane (*58*, *59*) today. Among known biochemical pathways, this congruence between geochemically catalyzed and enzymatic multistep reactions has no precedent. This anchors the origin of metabolism to the acetyl-CoA pathway via transition metal-catalyzed reactions of H_2_ and CO_2_ on mineral surfaces of serpentinizing hydrothermal systems (*47*), environments that have continuously existed on Earth since the existence of liquid water (*58*).

Among the four main sectors of metabolism—nucleotide synthesis, cofactors synthesis, amino acid synthesis, and central carbon metabolism—nucleotide synthesis stands out as enzymatically complete in LUCA (Fig. 2). This reflects its irreplaceable nature. The main function of nucleotides in cells is not small-molecule synthesis but transmission and expression of genetic information, functions that cannot be replaced by minerals and water. Several proposals for surface-catalyzed reactions that mirror biochemical nucleotide synthesis suggest a simple chemistry (*3*, *40*) yet involve ionic bonds of substrates to surfaces rather than the covalent carbon-metal bonds formed by native transition metals (*25*, *30*). By contrast, amino acid synthesis was incomplete in LUCA, requiring catalytic input from the environment and enzyme inventions in the lineages leading to LACA and LBCA (Fig. 2). In metabolism, amino acids are synthesized from 2-oxoacids via reductive aminations or transaminations (*60*). The same reactions are efficiently catalyzed by Ni^0^ (*28*), as are reverse tricarboxylic acid (rTCA) cycle reactions that generate biochemically relevant 2-oxoacid precursors from pyruvate and glyoxylate (*26*, *28*). These nonenzymatic reactions permitted continuous synthesis of simple amino acids, particularly ancient ones (*61*), during the assembly of metabolism before the origin of translation.

### Metabolic assembly uncovers independent enzyme origins

**Fig. 3.**
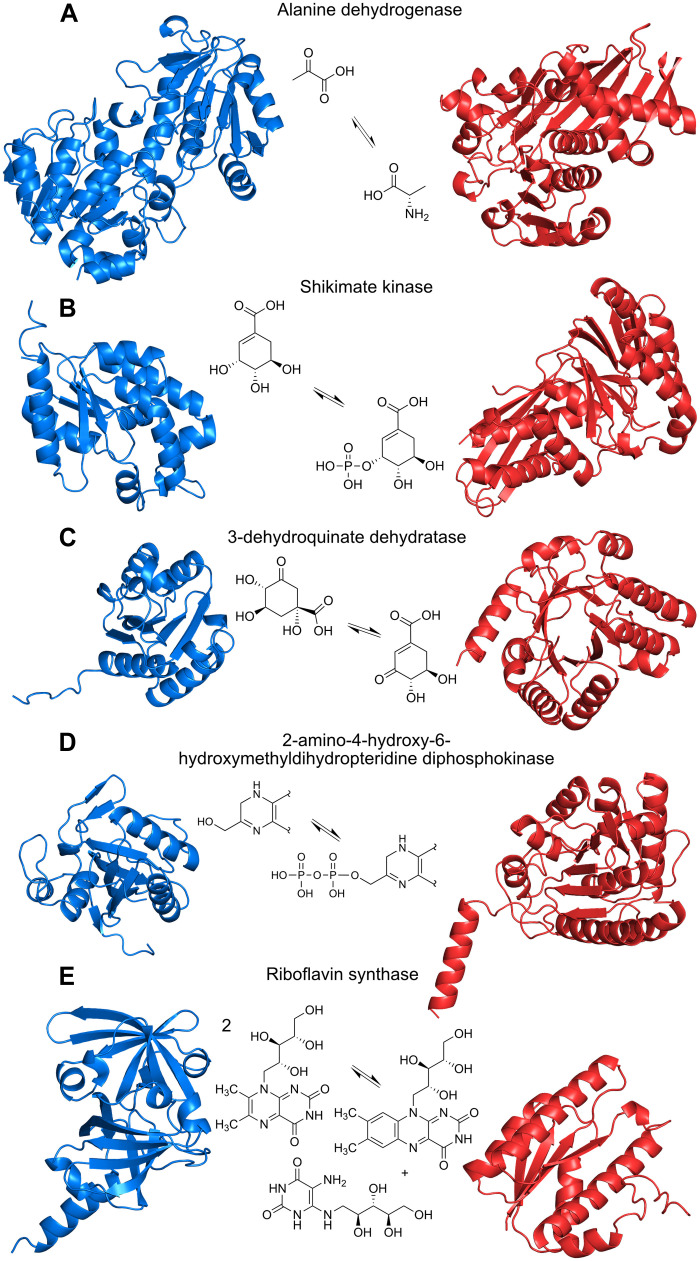
Protein families independently originated in the bacterial and archaeal domain. Five cases of independent origins for structurally unrelated protein families are shown: (**A**) AlaDH, (**B**) shikimate kinase, (**C**) 3-dehydroquinate dehydratase, (**D**) 2-amino-4-hydroxy-6-hydroxymethyl-dihydropteridine diphosphokinase, and (**E**) riboflavin synthase. The archaeal structure is in red, the bacterial structure is in blue, and a schematic of the catalyzed reaction is shown between them. The pairs each generate a TM-score of ≤0.5 in the pairwise comparison using US-align (see Materials and Methods).

The finding that metabolism underwent final assembly post-LUCA (Fig. 2) generates the prediction that independent origins of unrelated enzymes for the same reaction should be identifiable. We detected five such cases in which bacteria and archaea invented structurally unrelated enzymes to catalyze the same reaction, without the gene having undergone rampant transdomain transfer (*18*, *19*) subsequent to its origin. These examples are shown in Fig. 3. Alanine dehydrogenase [AlaDH; Enzyme Commission (EC) number 1.4.1.1] (Fig. 3A) catalyzes the reversible, NADH (reduced form of nicotinamide adenine dinucleotide)-dependent reductive amination of pyruvate to alanine (*62*). Independent origins of AlaDH in the LBCA and LACA lineages indicate that the environment provided this chemically facile function (*28*, *29*) in LUCA. Two enzymes of the shikimate pathway document post-LUCA innovations (Fig. 3): shikimate kinase and 3-dehydroquinate dehydratase. Archaea synthesize shikimate from different precursors than bacteria, although the shikimate kinase (Fig. 3B) and 3-dehydroquinate dehydratase (Fig. 3C) reactions are conserved (*63*) yet catalyzed by unrelated enzymes, suggesting a late enzymatic origin of aromatic amino acid synthesis (*61*). The shikimate pathway also leads to pterins, including tetrahydrofolate (H_4_F) and tetrahydromethanopterin (H_4_MPT), the methyl carriers in the acetyl-CoA pathway of bacteria and archaea. Also required for H_4_F and H_4_MPT synthesis, 2-amino-4-hydroxy-6-hydroxymethyl-dihydropteridine diphosphokinase (*64*) reveals independent origins en route to LBCA and LACA (Fig. 3D). In methyl synthesis of the acetyl-CoA pathway, two reactions (bacteria) or three reactions (archaea) are catalyzed by structurally unrelated enzymes (*56*), not listed here as independent innovations because the reactions are not identical, differing by the pterin cofactor H_4_F versus H_4_MPT, the syntheses of which themselves entail several unrelated enzymes (*55*). Riboflavin synthase involved in flavin synthesis (Fig. 3E) also reveals independent origins in LACA and LBCA, representing a case in which the reaction requires no catalyst (table S4), proceeding spontaneously at pH 7 upon boiling (*65*). Increased temperature increases reaction rate for most biochemical reactions (*48*), an observation favoring a thermophilic origin of metabolism.

### Metals generated metabolism in serpentinizing systems

The reactions surrounding the acetyl-CoA pathway and incomplete rTCA cycle take place in the presence of transition metals under serpentinizing hydrothermal conditions (*25*–*27*, *30*), but these pathways are not an exception in core metabolism. Among the reactions in our sample, 37 (10%) have been reported to proceed as in metabolism using the conditions and metal catalysts of hydrothermal vents (table S4). The metal catalysts (native and divalent transition metals) functionally replace enzymes and, in many cases, cofactors (table S4) in the laboratory, for example, NADH in carbonyl reductions to alcohols (*66*) or pyridoxal phosphate (PLP) in reductive amination of 2-oxoacids to amino acids (*28*, *29*). For a further 106 reactions, the reaction type but not the exact reaction has been shown, for example, reductive amination of carbonyls on alternative carbon backbones beyond the seven biological amino acid synthesis reactions demonstrated by Kaur *et al.* (*28*) (table S4). In 23 further reactions, the sequence from reactants to products proceeds under hydrothermal conditions, but the pathway in the laboratory involves a different number of chemical steps or a different reaction sequence. These abiotically catalyzed metabolic reactions mainly reside in carbon metabolism: the acetyl-CoA pathway (*25*, *27*, *30*), the incomplete reverse TCA cycle (*26*, *28*), glycolysis/gluconeogenesis (*67*), and the pentose phosphate pathway (*68*). However, many are involved in amino acid biosynthesis, supplying amino acids for synthesis of the first proteins.

While abiotic organic synthesis in modern serpentinizing systems is so far limited to the detection of formate (*58*, *59*), methane (*69*), and, more rarely, amino acids (*70*, *71*), serpentinizing systems are typically saturated with microbial life (*58*, *59*), often with an abundance of primary producers that use the acetyl-CoA pathway for carbon and energy metabolism, acetogens, and methanogens (*58*, *72*), such that abiotic organics observed in effluent samples (*58*) are typically leftovers of microbial growth. One particularly important property of hydrothermal systems for the origin of metabolism is their microporous structure, providing the ability to concentrate organic reaction products at or near their site of synthesis, thereby enhancing further reaction (*39*). The catalysts, reactants, reactions, and properties of serpentinizing hydrothermal vents have far-reaching congruence with the reactions of metabolism itself, suggesting that they represent the site, and the chemistry, of metabolic assembly.

### Phosphite is the source of phosphorylation during assembly

Under the conditions of serpentinizing hydrothermal vents, 91% of metabolic reactions in our sample are exergonic in the biosynthetic direction (fig. S2 and table S5), whereby 213 (59%) involve reactions of highly polarized, hence reactive carbonyl groups and 134 reactions (37%) involve reactions of phosphate groups (table S3). In modern metabolism, ATP is the currency of chemical energy that enables endergonic reactions to go forward (*9*, *21*). In cells that use the acetyl-CoA pathway for carbon and energy metabolism, ATP is synthesized by the rotor stator ATP synthase, requiring ion-tight membranes and mechanisms that couple ion pumping to the exergonic reaction of H_2_ with CO_2_ (fig. S3, table S6, and Supplementary Text). The source of high-energy phosphate bonds at metabolic origin, before the origin of membranes, is still discussed (*22*, *73*) because geochemically viable, continuous aqueous sources of phosphorylation have not been reported. Although reaction networks lacking phosphate in reactants and cofactors can be constructed using computers (*24*), the metabolic synthesis of most amino acids, all cofactors, and all nucleotides involves formation or cleavage of phosphate bonds—phosphate is a non-negotiable component at the origin of microbial metabolism. Acetyl phosphate, a possible primordial currency of high energy bonds (*39*), spontaneously forms from reactions of thioacetate and phosphate in water (*74*), although no continuous environmental source of thioacids is known. The growth of metabolism from a simple intermediate state (Figs. 1B and 2) prescribes a single environment for metabolic assembly (*40*, *49*), predicting the existence of a mechanism of phosphorylation that operates under serpentinizing conditions. In that search, microbial physiology informs.

Some bacteria can use phosphite (H_2_PO_3_^−^; P^+3^) as their sole source of phosphorus via the reaction H_2_PO_3_^−^ + AMP + oxidized form of nicotinamide adenine dinucleotide (NAD^+^) → adenosine 5′-diphosphate (ADP) + NADH (*34*, *35*), and some even use phosphite as their sole source of electrons for CO_2_ reduction via acetogenic carbon and energy metabolism involving the acetyl-CoA pathway (*34*). Shortly after the initial isolation of phosphite-dependent microbes (*35*), Buckel (*13*) raised the possibility that phosphite oxidation could have been a source of substrate-level phosphorylation in prebiotic evolution. Pasek *et al.* (*12*) subsequently reported that three of nine serpentinizing systems sampled and three of four that contained detectable phosphate contained detectable phosphite, while Frouin *et al.* (*75*) reported that the microbiota of serpentinizing systems are rich in phosphite oxidizing genes. Boden *et al.* (*76*) extended the observations of Frouin *et al.* (*75*) regarding sources of phosphite for microbial growth. Similar to Buckel (*13*), Mao *et al.* (*34*) suggested that the activity of AMP-dependent phosphite dehydrogenase could reflect a very ancient phosphorylation route, noting its occurrence in anaerobic autotrophs that use the acetyl-CoA pathway.

**Fig. 4.**
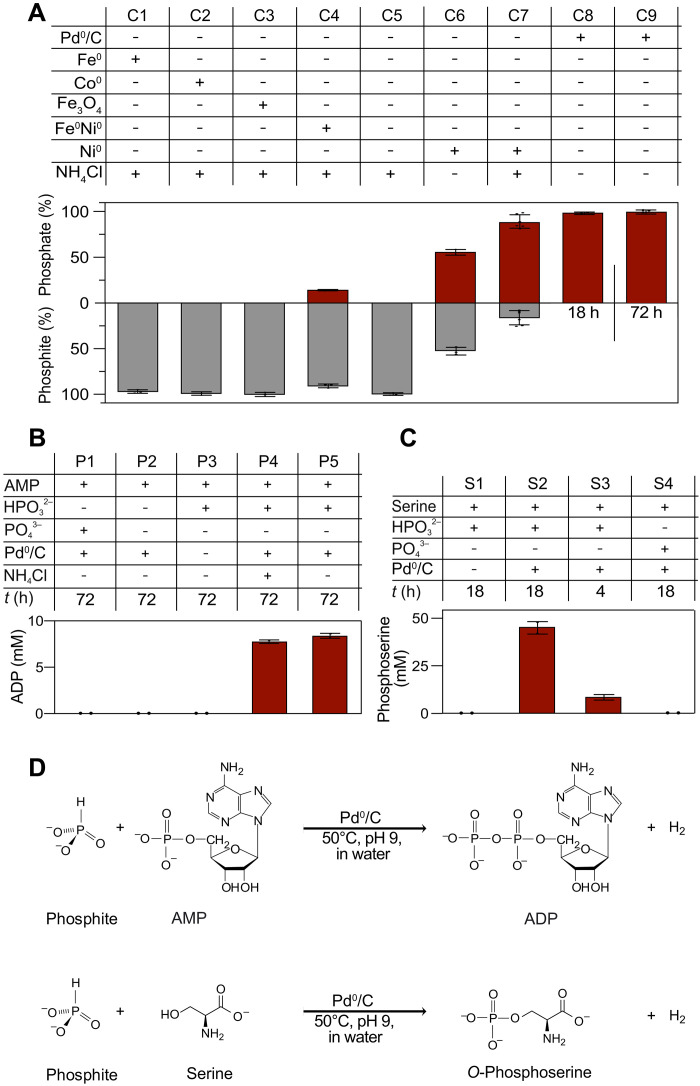
Aqueous, phosphite-dependent phosphorylation over metals. (**A**) Oxidation of 200 mM phosphite to phosphate under the conditions shown in the table (C1 to C9). Reactions were carried out at pH 9 and 100°C for 18 hours (h) (or 72 hours for C9), with or without 75 mM NH_4_Cl (*23*). The corresponding ^31^P NMR spectra are shown in fig. S4 (conditions C1 to C9). (**B**) Phosphite phosphorylates AMP (100 mM) to ADP over Pd/C in water at pH 9 and 50°C after 72 hours (spectra shown in fig. S5, P1 to P5). (**C**) Phosphite phosphorylates serine (100 mM) to phosphoserine over Pd/C at pH 9 and 50°C after 4 and 18 hours (spectra shown in fig. S5, S1 to S4). Use of phosphate instead of phosphite yields no detectable phosphorylation by ^31^P NMR (conditions P1 and S4). (**D**) Reaction of HPO_3_^2−^ with AMP and serine. Phosphite oxidation generates H_2_; the turnover number for phosphite per Pd atom was 2, but only a fraction of Pd is catalytically available, and only a portion of phosphate-producing reactions phosphorylate AMP or serine. The yields plotted in (A), (B), and (C) are an average of three reactions (C1 to C7 and P1 to P3) or two reactions (C8 and C9, P4 and P5, and S1 to S4). Each bar plot shows the results of a reaction under the set of conditions listed in the column above it. In all tables, the presence or absence of the chemical species listed in the rows is marked with “+” or “−” for each experiment (column). Further experimental details are given in table S7.

Although naturally occurring in serpentinized rocks (*12*), phosphite is widely discussed as, but not demonstrated to be, a prebiotic source of organophosphate bonds (*12*, *13*). It is a very strong reductant, with a standard midpoint potential of −690 mV (*35*) vastly exceeding that of H_2_ (−414 mV), but it is kinetically stable (*12*), meaning that it requires activation (*34*) to react. Because Ni^0^ is effective in activating CO_2_, H_2_, NH_3_, and organic molecules (*25*, *27*, *28*, *30*), we tested whether Ni^0^ could also activate phosphite. Across a broad pH range (from pH 9 to pH 11), phosphite in water is readily converted to phosphate and H_2_ by Ni^0^, but not by Fe^0^, Co^0^, or magnetite, at roughly 75% yields overnight at 100°C (Fig. 4A).

If we react 200 mM phosphite with 100 mM AMP, replacing AMP-dependent phosphite dehydrogenase (*34*) with Ni^0^ (1.5 mmol in 1.5 ml), then we observe after 96 hours at 50°C and pH 7 phosphorylation of AMP in water to 6.74 μM ADP, partitioned into three isomers, the ATP precursor ADP, adenosine 3′,5′-diphosphate, and adenosine 2′,5′-diphosphate (fig. S6). The ∼7 μM ADP yield demonstrates the unexpected ability of Ni^0^ to promote phosphorylation, but the yield is low; hence, we tested its group 10 homolog, palladium. Similar to Ni, elemental Pd is an efficient catalyst of TCA cycle reactions and amino acid synthesis (*28*) and is naturally deposited in serpentinizing systems, often as a component of awaruite (Ni_3_Fe) (*32*, *33*), which catalyzes CO_2_ fixation (*25*). In the presence of Pd (10% on carbon, Pd/C), phosphite in water phosphorylates the phosphate group of AMP at pH 9 to ADP with ∼8% yield in 72 hours at 50°C (Fig. 4B). The ∼8 mM concentration of ADP obtained with Pd/C is intermediate between the physiological concentrations of 560 μM ADP and 9.6 mM ATP in exponentially growing *Escherichia coli* cells (*77*). Phosphite over Pd/C also converts serine to phosphoserine, with 42% yield in 18 hours at pH 9 (Fig. 4C). Other biological substrates are also phosphorylated under these hydrothermal vent conditions, including acetate to form acetyl phosphate (*78*). In contrast to previous prebiotic phosphorylation protocols, no ammonium, cyanide, urea, or other condensing agents [discussed in (*22*, *23*, *73*)] are required for these metal-promoted phosphite-dependent phosphorylations, although 75 mM ammonium does not inhibit the reaction (Fig. 4B). These findings argue strongly in favor of a single, aqueous, phosphorylating environment for metabolic origin. For metabolic assembly, continuous, physiological-level phosphorylation in a hydrothermal system over hundreds of millennia is more life-like and more biosynthetically useful than intermittent spikes of phosphides from meteoritic delivery (*22*), because metabolic assembly required protracted geological time, continuous energy release, continuous phosphorylation, and a continuous environment for enzymatic invention (Fig. 2).

The concentrations of phosphite used in the present experiments are higher than those measured in serpentinized rocks, although phosphite concentrations have only been reported for a handful of serpentinizing systems (*12*). However, as Pasek *et al.* (*12*) point out, high temperatures and low water/rock ratios shift modeled equilibria with phosphate in favor of phosphite. In addition, the serpentinization process consumes water via reactions with host rock to an extent that host rock volume increases by up to 44% (*79*). This generates microfractured (increased surface area) environments and locally low water activities, hence higher solute concentrations, at the micrometer scale (*80*). Furthermore, the reactions of phosphite at metabolic origin took place ∼4 billion years ago in a geochemical setting where host rocks for serpentinization were more widespread than today (*81*). These factors, in addition to continuous aqueous phosphite and native metal supply through serpentinization over geological time scales, as opposed to our overnight laboratory reactions, weigh in favor of the case for a phosphorylating hydrothermal vent.

Native nickel emerges as a remarkably multifunctional, broad-spectrum catalyst for the incorporation of H_2_, CO_2_, NH_3_, and now (although less effective than Pd) phosphorus into nascent and primordial metabolism. As Ni^2+^, nickel is present in the active site of ancient enzymes of acetogens and methanogens: hydrogenases (*82*), carbon monoxide dehydrogenase, and acetyl-CoA synthase of the acetyl-CoA pathway (*50*, *51*), as well as methyl-coenzyme M reductase in methanogenesis (*54*). As Ni^0^, it functionally replaces the entire acetyl-CoA pathway, catalyzing nonenzymatic CO_2_ reduction to pyruvate (*25*, *27*, *47*), TCA cycle reactions (*28*), reductive aminations in amino acid syntheses (*28*), and promoting the phosphite-dependent phosphorylation of phosphate and hydroxyl groups in AMP (fig. S6). The phosphorylation reaction is thermodynamically favorable because the free energy of phosphite oxidation to phosphate, HPO_3_^2−^ + H_2_O → HPO_4_^2−^ + H_2_(g) with Δ*G*° = −46.3 kJ mol^−1^ at pH 7 (*83*), is sufficient to generate a phosphoanhydride bond in ADP (*34*), without the participation of external energy sources. Phosphite activation involves hydride removal from phosphite. Although neither Ni^0^ nor Pd^0^ are oxidants, both are good hydride acceptors and excellent catalysts of reversible H_2_ synthesis (*84*). These findings show that metals naturally deposited in serpentinizing systems (Ni^0^ and Pd^0^) promote the oxidation of phosphite, which is formed during serpentinization (*12*), to phosphate in such a manner as to phosphorylate phosphate and hydroxyl groups at physiological temperature and pH in water. Native metals of serpentinizing vents transform H_2_, CO_2_, NH_3_, and HPO_3_^2−^ into phosphorylated organic compounds in reactions similar or identical to reactions of metabolism.

### Cofactors replace environmental catalysts

Cofactor synthesis is conspicuously incomplete in LUCA’s metabolism (Fig. 2). Although traditionally thought to precede enzymes in biochemical evolution and while effective in promoting reactions without enzymes (*14*, *37*, *40*), cofactors do not readily arise spontaneously in aqueous prebiotic-type reactions (*14*). They have complicated biosynthetic pathways in metabolism (*85*), their chemical synthesis in the laboratory has to be meticulously orchestrated (*45*), and, in metabolism, they are often required for their own enzymatic synthesis (*14*, *39*, *86*). In core metabolism, most cofactors do not act as catalysts; rather, they participate as reactants that transfer mass, chemical moieties, to or from substrates (*37*). Moreover, in the presence of native metals, recent reports show that several cofactors even accept donor moieties directly from metal surfaces, uncovering insights into the primordial assembly of metabolism.

For example, H_2_ in the presence of Ni^0^, Fe^0^, and Co^0^ converts NAD^+^ to NADH in water (*82*), and Ni^0^/H_2_ can replace NAD(P)H [reduced form of nicotinamide adenine dinucleotide (phosphate)] as a reductant for numerous redox reactions of core metabolism that involve hydride transfer (*28*, *29*). The synthesis of low potential reduced ferredoxin, required in five core metabolic reactions, involves a multienzyme system of flavin-based electron bifurcation and one-electron transfers in cells (*87*, *88*), but ferredoxin can be reduced with single electrons stemming from H_2_ and Fe^0^ in water (*89*). PLP performs catalysis in numerous aminotransferase reactions in metabolism (*14*, *29*, *60*) (table S3), is replaced by Ni^0^ or H_2_/Ni^0^ in the presence of NH_3_ (*28*) (fig. S7), and is reductively aminated to pyridoxamine with Ni^0^, H_2_, and NH_3_ in water under hydrothermal conditions (*29*). It has been previously shown that CO_2_ and H_2_ react over Ni^0^, Fe^0^, or Ni_3_Fe to produce formate, acetate, pyruvate, and methane, whereby the metal catalysts replace roughly 10 enzymes and 10 cofactors of the acetyl-CoA pathway (*47*). In those reactions, the molybdenum cofactor (MoCo), which is required for enzymatic formate synthesis from CO_2_ (*52*), is replaced by Ni/H_2_, as is thiamine pyrophosphate (TPP), required for enzymatic pyruvate synthesis in the pathway (*27*, *90*). The same is true for the C1 carriers H_4_F and H_4_MPT, which are required for methyl synthesis from H_2_ and CO_2_ (*25*) in bacteria and archaea (*55*), and the cobamide (Cob) cofactor (*55*) required for metal-to-metal methyl transfer in the acetyl-CoA pathway (*91*). In Ni_3_Fe-dependent methane synthesis (*25*), the alloy replaces the function of the Ni-containing tetrapyrrole F_430_ of methanogenesis (*54*).

Although this list of metal-catalyzed metabolic reactions is short, a general principle emerges: Native metals activate and transfer H_2_-, CO_2_-, and NH_3_-derived moieties to substrates, generating biochemical products of metabolism and thereby functionally substituting for essential cofactors of core metabolism under hydrothermal conditions. Furthermore, in cases experimentally studied so far, the cofactors interact specifically with metal surfaces to obtain the activated moiety that they transfer to the substrate: hydride in the case of NADH (*82*), single electrons in the case of ferredoxin (*89*), amino groups in the case of pyridoxal (*29*), and phosphate in the case of ADP (Fig. 4B). Native metals are simpler and older than cofactors and are functionally equivalent to cofactors for group transfers in cases studied so far.

### Cofactors replace metals but are synthesized by enzymes

**Fig. 5.**
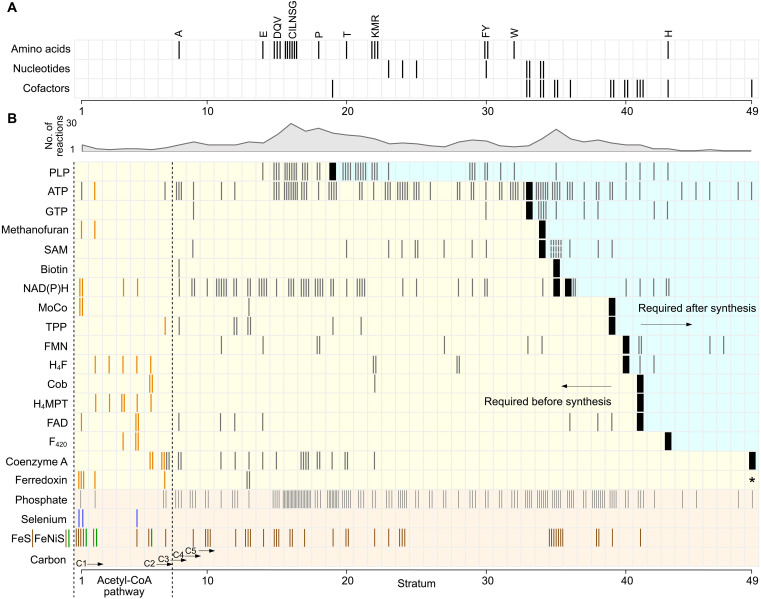
Serial order of metabolic products. Reactions were ordered by their appearance in metabolism (see Materials and Methods). (**A**) The appearance of amino acids (one letter code), nucleotides [from left to right uridine 5′-triphosphate (UTP), cytidine 5′-triphosphate (CTP), deoxy-CTP (dCTP), deoxythymidine 5′-triphosphate (dTTP), ATP/GTP, and deoxy-ATP/deoxy-GTP (dATP/dGTP)], and cofactors [order given in (B)]. See also Fig. 6. (**B**) The numbers of reactions appearing in each stratum (strata 1 to 49) are plotted. The matrix shows the order of appearance of reactions that require the given cofactor (narrow vertical lines) and reactions that synthesize the cofactor (solid vertical lines). Strata before the synthesis of the cofactor are shown in yellow, while strata following the synthesis of the cofactor are in blue. Vertical lines in sepia indicate reactions of the acetyl-CoA pathway, which are restricted to strata 1 to 7 (dashed lines). Ferredoxin is a protein with FeS clusters synthesized by ribosomes, not metabolism. Coenzyme B, coenzyme M, and F_430_ are not included because they are not involved in biosynthetic metabolism. Five of the six ATP-dependent reactions at stratum 34 correspond to donations of adenosyl or AMP residues. The order of amino acids is the order of their appearance in metabolism catalyzed by enzymes, recalling that the first enzymes were synthesized from nonenzymatically formed amino acids. References to metal-catalyzed reactions that serve as evolutionary precursors to cofactor-dependent reactions are given in fig. S8. Bottom: Reactions involving phosphate bonds, reactions catalyzed by selenoproteins, reactions catalyzed by proteins with FeS or FeNiS clusters, and first appearance of C1 to C5 carbon backbones. FMN, flavin mononucleotide.

Biosynthetic pathways should hold clues about the order in which catalysts arose (*14*, *40*). To address this issue systematically across core metabolism, we serially ordered the appearance of all reactions during metabolic assembly, starting at the acetyl-CoA pathway and leveraging the principle that the appearance of a reaction requires the existence of its reactants as products of earlier reactions (see Materials and Methods and Supplementary Text). For reactions that require cofactors as reactants, their metal-catalyzed moiety-donating equivalents were coded as being provided by the environment until the cofactor was synthesized, at which point it replaces the metallic forerunner. Because a metabolic network can grow by one reaction at several nodes simultaneously, reactions of equal age are grouped into the same time unit, or stratum, whereby the ensuing order and occupancy of strata are unique. The ordered list of enzymatic reactions in metabolism is given in table S8. The order in which products arise is of interest, in particular, the relative timing of cofactor synthesis. As expected, amino acids arise first, followed by nucleotides, consistent with their inferred synergistic interactions at origins (*92*), with cofactors emerging last (Fig. 5A). Altogether unexpected, however, was our finding that cofactors not only arise late but that all cofactors are required in reactions that precede their synthesis in metabolism (Fig. 5B). For 9 of 17 cofactors, the cofactor is synthesized in the last reaction of core metabolism in which it occurs. In total, cofactors are required for 209 reactions that precede their synthesis as opposed to 90 reactions that follow their synthesis. Moreover, 12 of 17 cofactors, including the last cofactor synthesized—CoA—are required in the first seven reactions at the starting point of metabolism: the bacterial and archaeal versions of the acetyl-CoA pathway (Fig. 5B). This organization of metabolism seems staggeringly illogical; it demands a simple explanation.

As one possibility, there could have existed more ancient alternative syntheses (*15*) for each cofactor, using starting compounds not found in biology, for example, in the case of cyanide-dependent synthesis of CoA (*45*), that might have provided organic placeholders for the biosynthesized cofactor at 23 different reactions up until its synthesis via enzymatic metabolism at stratum 49 (Fig. 5B). Once CoA became synthesized by CO_2_-dependent metabolism, the ancestral cyanide-derived CoA would no longer have been needed, although a cyanide-dependent synthesis cannot have coexisted with metabolic CO_2_-dependent CoA synthesis because the acetyl-CoA pathway is inhibited by cyanide (*93*). Although these possibilities are entertained (*15*, *45*), we consider that in laboratory reactions, transition metals can catalyze thioester bond formation and cleavage using simple organic thiols, such as alkyl sulfides, which exert the salient function of CoA (*4*), or catalyze the entire acetyl-CoA pathway (*25*, *27*, *30*, *47*). Why should metabolism have required a complicated, highly orchestrated, highly specific, abiotic synthesis of many (identical) molecules of CoA continuously over geological time if ubiquitous environmental thiols and metals can fulfill the function? The far simpler, metal-catalyzed, thiol-dependent reaction can, in principle, serve instead of the cofactor until the latter is synthesized by metabolism.

The same principle comes to bear for all other cofactors. For example, NAD(P)H is required in 47 reactions preceding its synthesis (Fig. 5B) but can be replaced (*82*) by H_2_/Ni^0^ in 29 reactions of metabolism (table S4), including reductive aminations (*28*); NADH (C_21_H_27_N_7_O_14_P_2_) exerts the function of irreducibly simple compounds, H_2_ and Ni^0^, present in the environment. H_4_F (C_19_H_23_N_7_O_6_) is required for one-carbon transfers in five reactions of the acetyl-CoA pathway (Fig. 5B), but its methyl synthesis and transfer function are replaced by H_2_ over Ni^0^ or Ni_3_Fe during aqueous pyruvate formation from CO_2_ (*25*, *27*, *30*). Metabolic assembly did not require abiotic H_4_F for pyruvate formation; H_2_ over native metals was sufficient. PLP is required in 15 reactions before its synthesis (Fig. 5B), but H_2_/Ni^0^ or Ni^0^ alone in the presence of NH_3_ can replace the cofactor (*28*) (fig. S7) in 21 laboratory metabolic reactions (table S4) and reductively aminate it to the active pyridoxamine form (*29*). As a final example, the MoCo (C_10_H_12_MoN_5_O_8_PS_2_), or its tungsten homolog Wco (C_20_H_22_WN_10_O_16_P_2_S_4_), is required for formate synthesis from CO_2_ in the first step of the acetyl-CoA pathway in bacteria and archaea but is also replaced by H_2_ over Ni^0^, Co^0^, Fe^0^, or Ni_3_Fe (*25*, *27*), all of which are naturally deposited in serpentinizing hydrothermal vents, which also provide H_2_ as reductant (*31*). Metabolic assembly did not have to await the synthesis of an organic precursor to MoCo/Wco to make formate, which is still synthesized today in serpentinizing hydrothermal vents (*58*, *59*), nor did metabolism have to await the synthesis of NADH, F_420_, reduced flavin adenine dinucleotide (FADH_2_), or reduced ferredoxin for reductions, TPP for pyruvate synthesis from H_2_ and CO_2_, or ATP as a source of phosphorylation (Fig. 5) because native metals and hydrothermal chemical constituents alone perform the reactions and phosphite-dependent phosphorylations conserve energy in the form of high-energy bonds. These findings are at odds with the view that cofactors are remnants of a hypothetical RNA world that existed before enzymes (*94*). Rather, cofactors appear as organic replacements for broad spectrum metal catalysts; enzymatically synthesized, they liberated primordial metabolism from its dependence on solid-state metals that fostered its assembly.

**Fig. 6.**
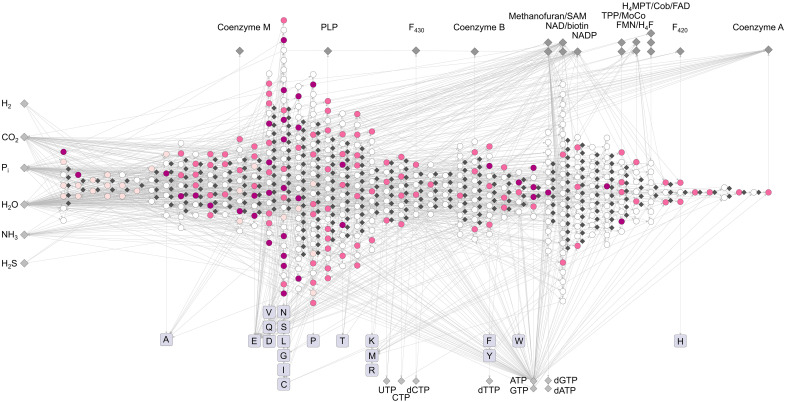
Structure of metabolic assembly. Network of reactions stratified as in Fig. 5. Inorganic substrates are shown at left. Network end products are shown at top (cofactors) and at bottom (nucleotides and amino acids indicated with one letter codes). Diamond-shaped nodes indicate chemical compounds; circular nodes indicate reactions. Reactions are shaded according to their classification in table S4: (i) (dark) The reaction has been experimentally shown to proceed catalyzed by transition metals or uncatalyzed, (ii) (light shading) the reaction is part of a pathway or reaction sequence from biological substrates to products catalyzed by transition metals, (iii) (intermediate shading) the reaction type (for example transamination) has been shown to proceed catalyzed by transition metals in water, or (iv) the nonenzymatic reaction is not yet experimentally demonstrated (white circles).

At current count, 46% of core metabolic reactions and geochemical analogs have been shown to take place without enzymes (and cofactors) under aqueous conditions of hydrothermal vents and transition metal catalysis (table S4), indicated as filled nodes in Fig. 6. The early synthesis of the Ni-containing tetrapyrrole F_430_ required in the last step of methanogenesis may seem unexpected given the late synthesis of Co-containing cobamides required in the acetyl-CoA pathway (Fig. 6). The difference stems from the synthesis of the lower ligand in cobalamin (*56*), which is lacking in F_430_. Cofactors that contain an AMP moiety such as NAD, flavin adenine dinucleotide (FAD), SAM (*S*-adenosyl methionine) or CoA arise subsequent to the synthesis of ATP, underscoring the requirement for an environmental source of phosphorylation at metabolic origin—HPO_3_^2−^ with Pd/C is such a source.

### Limitations of the study

Identification of archaeal functions is a limitation in current data. Many archaeal enzymatic functions in core metabolism are not yet known (*63*, *85*). Our analysis was restricted to enzymes of metabolism, hence limited to clusters having annotations of known biochemical function in at least one archaeal lineage. With expanded knowledge of functionally characterized enzymes of biosynthetic pathways in archaea, many more archaeal and archaeal-specific enzymes (*63*, *85*) will likely be uncovered. This results in an apparent excess of bacterial specific inventions. In addition, archaeal lineages are less well sampled than bacterial, compounding the same sampling related problem. The list of experimentally demonstrated environmentally catalyzed reactions at origins is still incomplete. Metals will likely not replace all of these enzymes, and field catalysts (*95*) will likely be required in many instances.

A critic might contend that the abundance of Pd in the crust, 1.5 × 10^−8^, is too low for relevance at origins, but physiology informs otherwise. Selenium is just as rare as Pd, 5 × 10^−8^, but despite crustal paucity, Se became incorporated into the primordial genetic code as selenocysteine. Moreover, three reactions of the acetyl-CoA pathway are catalyzed by selenoproteins (Fig. 5B) (*96*), indicating that elements as rare as Se were continuously available in the environment where metabolism and the genetic code arose. Although Pd^0^ is enriched in serpentinizing environments (*32*, *33*), it is still generally rare (*97*), and it typically occurs in the nickel-iron alloy awaruite (*32*, *33*, *97*). Nickel also usually occurs as awaruite in serpentinizing systems, whereby awaruite is also a very effective catalyst in laboratory CO_2_ fixation to prebiotic intermediates (*25*, *27*). On the Hadean Earth, Wilson cycles had not yet returned all primordial crust to the mantle for remelting and differentiation; hence, the frequency of Pd^0^ in primordial crust may have been higher than today. Future work will investigate the ability of PdNiFe alloys to catalyze carbon reduction and phosphite-dependent phosphorylation.

### Cofactors freed metabolism from the crust

If the 17 cofactors summarized in Fig. 5 and required in Fig. 6, all of which including ATP have metal-dependent functional precursors, were not needed at the geochemical onset of metabolism, then why were they needed at all, and why in the terminal phases of metabolic assembly via enzymes? The answer, we propose, is that the cofactors were required as a chemical replacement for the native metals that enabled metabolic assembly, hence cofactor synthesis from the outset, such that metabolism could become independent of solid-state catalysts deposited in Earth’s crust. By severing the connection between metabolism and environmental catalysts, enzymatically synthesized cofactors rendered metabolism autocatalytic—generating its required catalysts by itself. The implication is that, in the extreme, the highly conserved organic cofactors of metabolism did not exist in their present form before the origin of ribosomal protein synthesis, which does not directly require cofactors other than guanosine 5′-triphosphate (GTP) (*42*). This would not preclude the existence of structurally simpler but functionally equivalent precursors of cofactors, which later became replaced (*40*, *98*).

**Fig. 7.**
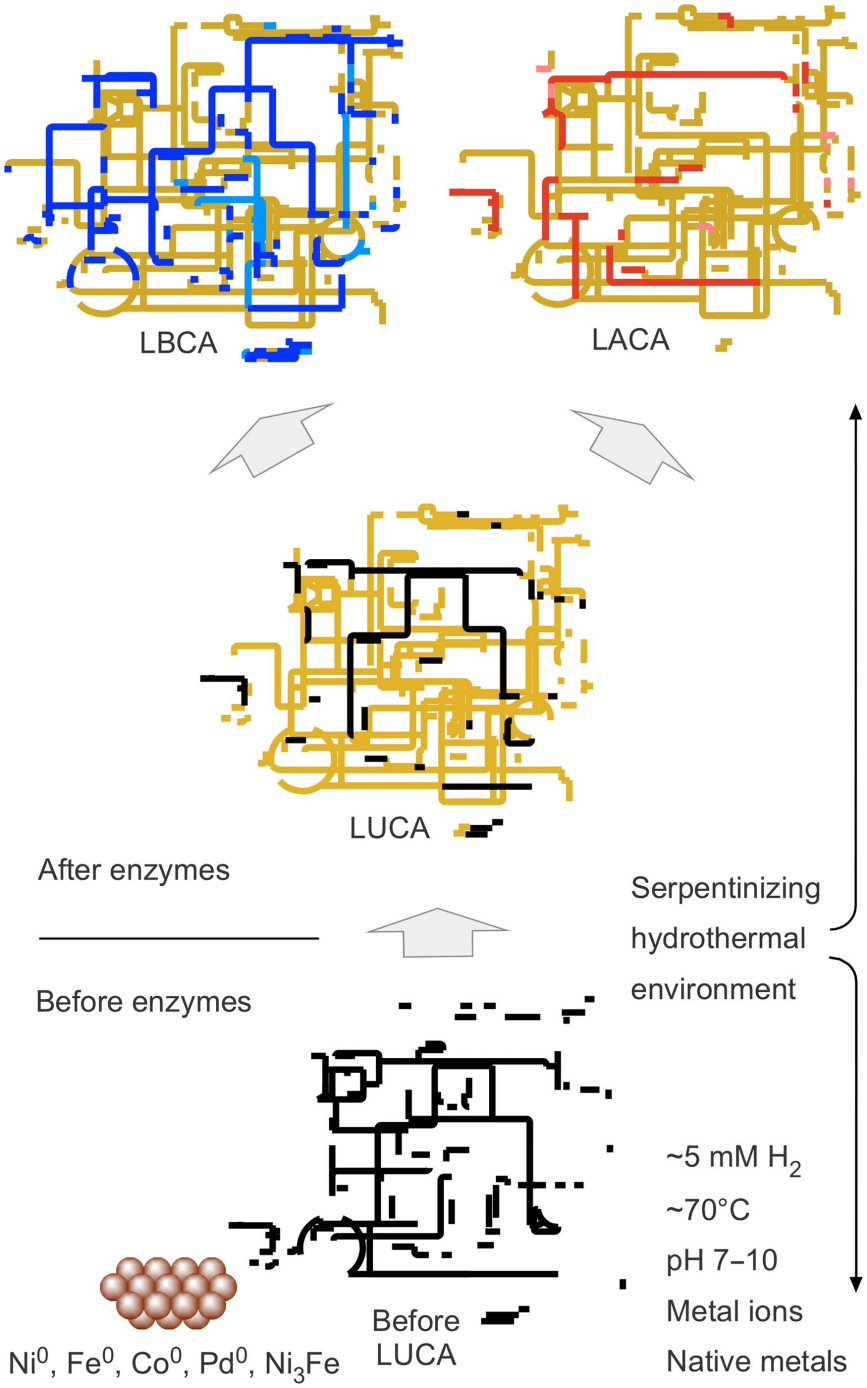
Assembly of metabolism at a phosphorylating hydrothermal vent. Maps prepared with iPath (see Materials and Methods) show reactions catalyzed by enzymes present in LUCA (gold) and lineage-specific additions in LBCA (blue) and in LACA (red). Lighter-shaded reactions in LACA and LBCA indicate enzymes in gray areas of Fig. 1B. Black lines denote reactions that are either (i) present as enzymatic in LUCA, LBCA or LACA and can be catalyzed by native or divalent transition metals (see fig. S8 and table S4) before enzymes (lower panel) or that are (ii) present as enzymatic in either LBCA or LACA, but not in LUCA, and can be catalyzed by native or divalent transition metals in LUCA. Catalysis is provided by enzymes and native metals (symbolized at bottom left), which catalyze one-electron transfer, hydride transfer, H_2_ activation, CO_2_ reduction, C1 transfer, formate synthesis, acetate synthesis, pyruvate synthesis, aldol condensation, decarboxylation, amino acid synthesis, and amination (see fig. S8), in addition to phosphite activation and phosphorylation (Fig. 4).

The gradual assembly of metabolism from environmental reactions to reactions present in LUCA and the independent growth of metabolism in the lineages leading to LBCA and LACA is outlined in Fig. 7. As metabolism underwent assembly, enzymes and cofactors gradually replaced metals and reaction rates (an unwieldy variable for environmental metals) came under the control of natural selection. Enzymes and cofactors provided the substrate specificity (*99*) required for metabolism to operate as a unit and fostered its hallmark: All reactions proceed at roughly the same rate (*48*) so that carbon and energy metabolism could form a tightly connected, stoichiometrically balanced (*87*) reaction. Given that native metals (Co^0^, Ni^0^, Fe^0^, and Pd^0^) are excellent prebiotic catalysts, performing the reactions of 17 different cofactors (fig. S8), why did they not become incorporated into enzymes during metabolism? Their specificity is too low, and they catalyze too many different reactions well (*100*) (fig. S8), making them a poor choice for prosthetic groups of enzymes.

Many theories for metabolic origin posit that FeS minerals in the environment served as catalysts before the origin of enzymes (*3*, *39*, *40*), yet FeS clusters of modern proteins serve mainly as mediators of electron transfer (*52*). The present findings indicate that the essential catalysis of origins was exerted not by metal sulfides but by native metals deposited in serpentinizing hydrothermal systems (*31*–*33*). During metabolic assembly, they were replaced by enzymes and cofactors, the latter serving as soluble replacements for native metals in the rock-and-water environment where metabolism and cofactors arose: a H_2_-producing and phosphorylating hydrothermal vent.

## MATERIALS AND METHODS

### Data acquisition

Prokaryotic whole-genome protein sequences were downloaded from the RefSeq database (release 223, May 2024). The data were filtered for the largest genome per species for archaea and the largest genome per family for bacteria. Subsequently, the list of acetogens without cytochromes from Rosenbaum and Müller (*101*) was checked for organisms (exact species and strain) not present in the filtered dataset but whose genomes were present in the downloaded complete RefSeq dataset. These were added to our filtered dataset to account for underrepresentation. Genomes with a size larger than 1000 proteins were retained (to exclude parasites). This yielded a balanced dataset of 401 archaeal and 552 bacterial genomes (see the “Data, code, and materials availability” section). In-house Python scripts, in some cases modified and debugged with ChatGPT 4o and Deepseek V3 and R1, were used for data processing throughout this work.

Reactions and EC numbers for core metabolic reactions that generate nucleotides, cofactors and amino acids from CO_2_, H_2_, NH_3_, H_2_S_,_ and P_i_ were obtained from Wimmer *et al*. (*17*). EC numbers were updated according to Kyoto Encyclopedia of Genes and Genomes (KEGG; release 112.0, November 2024). EC numbers for enzymes involved in archaeal and bacterial glycolysis and gluconeogenesis were obtained from Bräsen *et al.* (*102*), the reaction IDs were retrieved from KEGG, and this reaction set was added to the list of core metabolic reactions. The archaeal H_4_MPT-dependent variant of the Wood-Ljungdahl pathway methyl branch (from KEGG module M00567) was also added, resulting in an updated list of 424 core metabolic reactions (table S9). Reactions added in this study were polarized in the biosynthetic direction, except for irreversible glycolytic reactions [based on Bräsen *et al.* (*102*)].

### Clustering and annotation of protein families in the biosynthetic core

Protein sequences from 953 prokaryotic genomes were clustered into protein families as described in Brueckner and Martin (*103*), with DIAMOND-blastp (*104*) used instead of BLAST. The results were filtered with an *e* value cutoff of ≤10^−5^ and a local identity cutoff of ≥25%. Hits with a global identity cutoff of ≥25% were used for Markov Chain Clustering. Of the resulting 245,258 clusters (see the “Data, code, and materials availability” section), only those containing 4 or more sequences were retained, resulting in 103,482 MCL clusters.

To annotate the clusters, bacterial and archaeal sequences corresponding to the EC numbers of 424 core metabolic reactions (table S9) were retrieved from SwissProt (release 2024_04) using the Application Programming Interface (API). MCL clusters were aligned using DIAMOND blastp against the SwissProt dataset, and the results were filtered for ≥40% sequence identity and an *e* value of ≤10^−5^. After filtering, each sequence in the clusters was annotated with the EC of its best hit, if there was one. The entire cluster was assigned an EC number when a minimum of 50% of its sequences were annotated with that EC.

For all EC numbers corresponding to the 424 core metabolic reactions that did not annotate a cluster, the corresponding prokaryotic sequences from UniProt (*105*) were downloaded through the API filtering for levels 1, 2, and 3 of protein existence evidence (to exclude predicted and uncertain proteins). Overall, database sequences for 369 (of 389) EC numbers were obtained. The annotation workflow was repeated as described above, now using the sequences downloaded from both SwissProt (release 2024_04) and UniProt (release 2024_06) as the target database. This yielded 1473 MCL clusters annotated with 335 EC numbers corresponding to 361 metabolic core reactions. The enzyme name was obtained from KEGG based on the EC.

### Merging of protein families in the biosynthetic core

MCL clusters that correspond to the same reaction (or at least one of the reactions they are mapped to is the same) were examined to determine if they could be combined. In the first step, sequence similarity–based merging was carried out. Clusters mapping to the same reaction were categorized into small (<50 members) and large (≥50 members) clusters. An all-versus-all DIAMOND blastp was performed to determine how many members of the small clusters matched the large ones and how many members of large clusters matched other large clusters. The cutoff was set to ≥25% sequence identity and an *e* value of ≤10^−5^. If more than 50% of sequences in a small or large cluster was above threshold in the alignment to a large cluster, then the clusters were merged (and with them, all reactions they map to) (fig. S1 and table S10).

In the second step, structural similarity was evaluated for clusters that correspond to the same reaction but could not be joined based on sequence similarity. A representative sequence for these clusters was chosen on the basis of its highest average local identity to all other cluster members out of the sequences with the highest number of BLAST hits in the cluster. Structures for the representative sequences were modeled with AlphaFold 2.2.0 (*106*) and ColabFold (*107*) or retrieved from the AlphaFold database (*108*). Representative structures of clusters mapped to the same reaction(s) were aligned using US-align (*109*). Template modeling (TM) scores normalized by the length of both sequences in the comparison were obtained, and if at least one of the two TM-scores was ≥0.5, then the structures were considered to share a common fold (*110*) (table S10), and these clusters were merged (fig. S1 and see the “Data, code, and materials availability” section). Clusters mapped to the same reaction could correspond to subunits of the same enzyme complex, so manual verification of subunit information was conducted on the basis of the sequences used for the annotation of each cluster.

### Assignment criteria for biosynthetic core protein families and Δ*G* calculation

The taxonomic distribution of each protein family (cluster) was calculated as the percentage of genomes in each higher taxon (archaeal orders and bacterial classes) that contain a member of that protein family. The artificial taxa “other bacteria” and “other archaea” were introduced for genomes from very small higher taxa containing less than four genomes or genomes with no information on taxonomic order/class. Clusters present in <10% of archaeal and <10% of bacterial genomes were classified as rare protein families that provide limited information and were not considered further (listed in table S2). The distribution table with the remaining clusters was sorted by the quotient 15 × (sum of bacterial distribution percentages in the cluster)/26 × (sum of archaeal distribution percentages in the cluster), where 15 is the number of archaeal and 26 is the number of bacterial higher taxa. Zero denominators were replaced with 1. The table was sorted left-to-right on the basis of increasing gene frequency per column (taxon) in bacteria and decreasing gene frequency per column (taxon) in archaea.

The following gene retention criteria were applied: If the gene (cluster) was retained in ≥50% of genomes of ≥4 bacterial, but not archaeal higher taxa, then it was classified as LBCA. If, on the other hand, it was retained in ≥50% of genomes of ≥4 archaeal, but not bacterial higher taxa, then it was classified as LACA. A cluster was categorized as tracing to LUCA if it occurs in ≥50% of genomes of ≥4 taxa in both bacteria and archaea. The LBCA and LACA clusters were resorted on the basis of the total percentage of bacterial and archaeal members, respectively. These conservative criteria of gene retention resulted in two uncertain regions that include clusters that are predominantly bacterial but do not satisfy the LBCA criteria (the gray LBCA region) and those that are predominantly archaeal but do not satisfy the LACA criteria (the gray LACA region) (table S2). Clusters that did not satisfy the gene retention criteria were manually moved to the gray categories, resulting in the distribution in Fig. 1B and table S2.

Cluster 692, corresponding to AlaDH, is a borderline case because it is present in ≥50% of genomes of four bacterial classes, half of which contain a small number of genomes: four in Myxococcia and six in Thermoleophilia, with the protein family present in 50% of both. This distribution and previous work reporting the unrelatedness of the archaeal and bacterial enzyme (*111*) classify cluster 692 as independently arisen in Fig. 3.

The taxonomic distribution data were visualized in a matrix (Figs. 1B and 2 and fig. S1) with the Python packages Matplotlib, Pandas, and Seaborn. Protein structures in Fig. 3 were rendered with PyMOL (The PyMOL Molecular Graphics System, version 2.5.4., Schrödinger LLC). Metabolic maps for Fig. 7 were prepared with iPath (*112*). Reactions corresponding to protein families assigned to LUCA, LBCA, and LACA (table S2) and reactions that could have an environmental precursor (table S4) were visualized. A caveat is that the tool could not display some KEGG reactions in the maps. Values for Δ*G* under different conditions (fig. S2 and table S5) were calculated as described in Wimmer *et al*. (*17*). Literature references are listed for manually curated information (table S4; enzyme references and enzyme promiscuity assignment in table S2). Chemical reaction types in table S3 were manually assigned.

### Gene distribution and structure visualization of ribosomal proteins

Protein families were not annotated with ribosomal proteins sequences, as they are not part of the autotrophic biosynthetic core. For ribosomal proteins (Fig. 1A and table S1), known to be short and biased in composition (*113*), all SwissProt prokaryotic ribosomal protein sequences of protein existence level 1 identified by protein name (table S1) were retrieved. These sequences were used as queries in DIAMOND blastp alignments against the balanced dataset of 401 archaeal and 552 bacterial genomes (see the “Data acquisition” section). Hits were considered homologs if they exhibited a local sequence identity ≥25% and an *e* value of ≤10^−10^.

Ribosomes from *E. coli* and *Thermococcus kodakarensis* are taken as models for a bacterial and an archaeal ribosome (Fig. 1A); therefore, ribosomal proteins from these ribosomes were extracted from the BLAST hits file filtering by ribosomal protein names (table S1), and a taxonomic distribution matrix was generated. Universal and domain-specific ribosomal proteins were identified on the basis of the literature (*114*–*116*). When BLAST searches were not sensitive enough to detect homologies known from the structural literature, rows were manually joined to reflect known evolutionary relationships. Figures of protein structures [Protein Data Bank IDs: 7K00 (*117*) and 6SKF (*116*)] were rendered with PyMOL (The PyMOL Molecular Graphics System, version 2.5.4., Schrödinger LLC). Domain-specific components were colored according to (*114*–*116*).

### Stratification of reactions and compounds

An upgraded version of the software CatReNet (v. 1.0.0) (*118*) used for the analysis is available at https://software-ab.cs.uni-tuebingen.de/download/catrenet/welcome.html. This release was designed to include a stratification algorithm based on the following formal framework [which extends concepts from Steel *et al.* (*119*)].

Let X be any set of molecule types that can arise by starting from a food set F of basic molecule types and repeatedly applying reactions from some given reaction set R. The number of possible orderings of the reactions that will generate all the molecule types in X could be astronomical; however, there is a well-defined underlying partial order on (i) the reactions and (ii) the elements of X. These two orders reveal how early any given reaction could possibly have occurred, and any given molecule type could have first appeared. Moreover, there is a fast way to compute these partial orders, and they are uniquely determined by the system (so multiple runs of the algorithm are unnecessary). We refer to this process as stratifying the sets R and X.

Formally, given reaction system with food set (X,R,F), where R is F generated, let $R_{0}=\left\{r\in R:\rho \left(r\right)\subseteq F\right\}$, where ρ(r) is the set of reactants of r. For i≥1, let

$$R_{i}=\left\{r\in R:\rho \left(r\right)\subseteq F\cup \pi \left(\underset{0\leq j<i}{⋃}R_{j}\right)\right\}$$

The sets $R_{i}$ are “nested” (i.e. $R_{i}\subseteq R_{i+1}$ for all i), and since R is F generated, $R_{i}=R$ for some value of i. For each. r∈R, we can then define an associated ranking λ(r) to be the smallest value of j for which $r\in R_{j}$.

We can also stratify X for any F-generated set R, as follows. Let $X_{0}=F$, and for each i≥1, let

$$X_{i}=X_{i-1}\cup \left[\underset{r\in R:\rho \left(r\right)\subseteq X_{i-1}}{⋃}\pi \left(r\right)\right]$$

Thus, $X_{i}$ is $X_{i-1}$ together with all the products of all the reactions in R that have all their reactants in $X_{i-1}$. The resulting collection of subsets of X thus forms a nested increasing sequence of molecule types: $F=X_{0}\subset \cdots \subset X_{K}=X_{K+1}$. Next, define $\lambda^{\prime }:\pi \left(R\right)\to \left\{1,2,\ldots \right\}$ by

$$\lambda^{\prime }\left(x\right)=\text{min}\left\{i:x\in X_{i}\right\}$$

In this way, the elements of π(R) are partitioned by their λ values, and $\lambda^{\prime }$(x) is a measure of how early element x can be generated (further technical details are provided in the Supplementary Text).

To determine the order of emergence of core metabolic reactions, the reaction set had to be adapted to form a continuous network (for a list of excluded and included reactions, see table S8). Cosubstrates such as NADH or reduced ferredoxin were included in the food set (for a full list, see table S8), as previous work has shown that their functions could be replaced by mineral surfaces at the origin of metabolism (see sections “Cofactors replace environmental catalysts” and “Cofactors replace metals but are synthesized by enzymes” and fig. S8). Compound recoding was carried out in those cases where it was necessary to distinguish between the environmental version (the function of the cofactor is carried out by mineral surfaces) and the synthesized, organic version of a cofactor or a product generated from it (table S8). The point of synthesis of a cofactor marks the start of its use in the network in place of the environmental precursor. All reactions that require a cofactor and occur after its synthesis naturally use the organic version, even if it is not specified in the recoding. The complete stratification results can be found in table S8.

To generate Fig. 5, the participation of most cofactors at a given stratum obtains from its occurrence in a given KEGG line reaction. Cofactors that are not listed in KEGG line reactions (for example, PLP-dependent transaminations) were retrieved from SwissProt (release 2025_03) via EC numbers associated with the reactions filtered for prokaryotic entries, with subsequent manual curation (table S8). If a target compound was synthesized in more than one reaction in the same stratum, then the synthesis was scored with only one bold line in the figure. When both NADH and NADPH could be used in the same reaction, only one occurrence was counted. Reactions that involve the formation or breaking of bonds with phosphate were scored on the basis of table S3.

To generate Fig. 6, the reaction network used in the stratification analysis (table S8) was converted into a GML file. Original, nonrecoded reactions were used, and H_2_, CO_2_, P_i_, H_2_O, NH_3_, and H_2_S were set as starting compounds for the figure. Network edges were defined from each reactant (diamond nodes) to the corresponding reactions (circular nodes) and from these reactions to the resulting product compounds (diamond nodes). When the product compound was a chemical moiety carried by cofactors such as H_4_F, H_4_MPT, methanofuran, or CoA, the cofactor-free, environmental variant was marked in the figure as a node in the corresponding stratum. The resulting network was visualized in Cytoscape (version 3.10.3) (*120*) and arranged in a left-to-right orientation according to the stratification defined in table S8. Annotation data from table S4 were integrated, with reactions highlighted in distinct color shades.

### Laboratory reactions and product identification

Reactions were performed as described in Schlikker *et al.* (*29*), unless otherwise stated. Reactors were pressurized with 5-bar argon (99.996%; Messer, Lenzburg, Switzerland). Before heating, reactors were sealed and purged with argon to remove dissolved and gaseous oxygen. No substantial difference in product conversion was detected between standard conditions and control experiments carried out in a glove box. Therefore, reaction mixtures were not prepared in a glove box.

#### Organic acid and amino acid reactions

Pyruvate, fumarate, 4-methyl-2-oxopentanoate, and glutamate (20 mM; Merck, Sigma-Aldrich, Darmstadt, Germany) were used as reactants with 0.6 mmol of catalyst in a total reaction volume of 1.5 ml. Ni-SiO_2_/Al_2_O_3_ (Merck, Sigma-Aldrich, Darmstadt, Germany) was used as catalyst in all reactions; SiO_2_/Al_2_O_3_ (Merck, Sigma-Aldrich, Darmstadt, Germany) was additionally included in glutamate cyclization experiments. Reactions were performed at 100°C and adjusted to pH 9 or pH 11. Reaction times were 1, 2, 4, or 18 hours. Products were identified by ^1^H nuclear magnetic resonance (NMR) spectroscopy at the Center for Molecular and Structural Analytics at the Heinrich Heine University Düsseldorf (CeMSA@HHU). ^1^H NMR spectra were recorded on a Bruker Avance III (600 MHz) spectrometer in a H_2_O/D_2_O mixture (6:1). Sodium 3-(trimethylsilyl)-1-propanesulfonate was used as the internal standard (CH_3_ peak at 0 parts per million) with a noesygppr1d pulse program. The relaxation delay (D1) was 1 s, with a time-domain size (TD) of 98,520 and a sweep width (SWH) of 12,315.271 Hz. We acquired 16 scans per sample. Integration was performed using Chenomx NMR suite (version 9.02). Results are shown in fig. S7 (for raw data, see “Data, code, and materials availability” section).

#### Phosphite activation experiments

Reactions were carried out with 200 mM sodium phosphite (Na_2_HPO_3_; Merck, Sigma-Aldrich) as the reductant. Where indicated, 200 mM ammonium chloride (NH_4_Cl; Merck, Sigma-Aldrich) was added. Catalysts included nano-nickel (Ni^0^), nano-iron (Fe^0^), cobalt powder (Co^0^), magnetite (Fe_3_O_4_) (all Merck, Sigma-Aldrich), a 1:1 (mol/mol) mixture of iron and nickel (Alfa Aesar) (1.5 mmol per vial), or Pd on activated charcoal (Pd/C) (Merck, Sigma-Aldrich; 1 mmol per vial, corresponding to 0.1 mmol of Pd). Reactions were performed at pH 9 for 18 and 72 hours with a total volume of 1.5 ml (1.0 ml for Pd/C reactions). Products were analyzed by ^31^P NMR spectroscopy at the CeMSA@HHU using a Bruker Avance III 600-MHz spectrometer. Spectra were recorded in proton-coupled and proton-decoupled modes in a H_2_O/D_2_O mixture (6:1) with a zgpg30 pulse program. The relaxation delay (D1) was 2 s, with a TD of 65,536 and an SWH of 96,153.844 Hz. We acquired 16 scans per sample. Spectra were processed with MestReNova (version 14.02) and are shown in fig. S4. Signal integrals were converted to concentrations using calibration curves obtained from phosphite and phosphate standard series. Results are shown in Fig. 4. 

#### AMP and serine reactions

Reactions with AMP (Merck, Sigma-Aldrich) contained 100 mM AMP and 200 mM sodium phosphite (Merck, Sigma-Aldrich, Darmstadt, Germany) or 200 mM sodium phosphate (Merck, Sigma-Aldrich, Darmstadt, Germany) as control. Selected experiments included 75 mM NH_4_Cl. Reactions were performed at pH 7 (nano-Ni^0^) or pH 9 (Pd/C) and 50°C with either 1.5 mmol of nano-Ni^0^ or 1 mmol of Pd/C as catalyst in a 1.5- or 1-ml reaction volume, respectively. Reaction times were 4, 18, or 72 hours (96 hours for Ni^0^ reactions). Standards of adenosine 5′-diphosphate disodium salt (ADP), adenosine 3′,5′-diphosphate disodium salt, and adenosine 2′,5′-diphosphate sodium salt were purchased from Merck (Sigma-Aldrich, Darmstadt, Germany). ADP formation in Pd/C reactions was analyzed by ^31^P NMR spectroscopy as described for phosphate activation experiments. Spectra were processed with MestReNova (version 14.02), and ADP concentrations were determined using calibration curves generated from ADP standards. ADP over nano-Ni^0^ was identified by electrospray ionization–liquid chromatography–mass spectrometry (ESI-LC-MS) (see below). Reactions with 100 mM serine (Merck, Sigma-Aldrich) were performed with 1 mmol of Pd/C in a 1-ml reaction volume and 200 mM sodium phosphite under the same conditions. Products were analyzed by ^1^H NMR spectroscopy as described for organic acid and amino acid reactions, and phosphoserine was identified and quantified using Chenomx NMR suite (version 9.02) with its internal compound library. Products were also analyzed by ^31^P NMR in coupled and decoupled modes as described for phosphite activation reactions, and spectra were processed with MestReNova (version 14.02). Data are shown in Fig. 4 and fig. S5.

#### ADP identification by ESI-LC-MS

ADP with Ni^0^ was identified using LC-UV-MS with a Dionex UltiMate 3000 UPLC system (Thermo Fisher Scientific, Dreieich, Germany) coupled to a maXis 4G (Bruker Daltonics, Bremen, Germany) quadrupole-time-of-flight mass spectrometer connected to an ESI ion source. Sample volumes of 10 ml were applied to a 3 mm–by–150 mm C18 XSelect HSS T3 column (2.5-μm particle size and 100-Å pore diameter; Waters, Milford, MA, USA) and separated using a binary gradient with a flow rate of 0.3 ml min^−1^. Mobile phase A was water + 0.1% formic acid, and mobile phase B was methanol + 0.1% formic acid. Starting with 5% B, a linear gradient to 95% B was applied from 2.5 to 15 min, followed by 95% B for additional 2 min and a return to 5% B within 1 min. The system was equilibrated with 5% B for another 4 min before the next injection. The UV detector was set to 254 nm, the wavelength at which adenosine absorbs. The MS (positive-ion mode) was run at a capillary voltage of 3.5 kV, a nebulizer pressure of 1 bar, a dry gas flow of 8 liter min^−1^, and a dry temperature of 200°C. Data acquisition was performed with Compass HyStar software (version 6.0.30.0, Bruker). ADP was identified and quantified from full-scan MS data (mass range, 50 to 1000 mass/charge ratio) using the Data Analysis (version 5.3) software (Bruker). Results are shown in fig. S6.

### Statistical analysis

The significance of the observed archaeal-bacterial distribution pattern was assessed with a two-sided permutation test from the SciPy Python package based on the difference in means of the proportion of genomes carrying the genes (column means in table S2) between bacterial and archaeal taxa, with 1 million resamples. A *P* value of <0.05 was taken as significant.

## Supplementary Materials

### The PDF file includes:

Supplementary Text Figs S1 to S8 Legends for tables S1 to S10 References
