# Supplementary material for: Intermediate stages in the origin of metabolism at a phosphorylating hydrothermal vent
Source: Sci Adv. 2026 Aug 5;12(32):eaef3128. doi: 10.1126/sciadv.aef3128 (PMC13440417; doi:10.1126/sciadv.aef3128)
Supplement: Supplementary file 1 [file sciadv.aef3128_sm.pdf]

Science Advances
AAAS

Supplementary Materials for

Intermediate stages in the origin of metabolism at a phosphorylating hydrothermal vent

Natalia Mrnjavac *et al.*

Corresponding author: Natalia Mrnjavac, n.mrnjavac@hhu.de

Sci. Adv. **12**, eaef3128 (2026)
DOI: 10.1126/sciadv.aef3128

The PDF file includes:

Supplementary Text
Figs S1 to S8
Legends for tables S1 to S10
References

Other Supplementary Material for this manuscript includes the following:

Tables S1 to S10

Supplementary Text

Reaction stratification: mathematical details

Definitions and initial results

A *reaction network* is a set X of molecule types, and a set R of reactions, each represented by writing $r: A \to B$ where A and B are nonempty subsets of X, referred to as the *reactants* and *products* of r (denoted $\rho(r)$ and $\pi(r)$, respectively). For a set R' of reactions, let $\pi(R')$ be the molecule types that are a product of at least one reaction in R'.

We also consider a particular subset of X called the *food set* F. A reaction system with a food set is denoted by the triple (X, R, F).

A subset R' of the reaction set R has an *admissible ordering* (or more briefly is *admissible*) if the reactions can be ordered in such a way that each reactant of each reaction $r \in R'$ is either present in the food set or is a product of an earlier reaction in the ordering. In the RAF (Reflexively Autocatalytic and Food set generated) literature (see e.g. (43, 119)), this notion is equivalent to the condition that R' is "F-generated".

Recall also the notion of the *closure* of the food set under a (sub)set R' of reactions, denoted $cl_{R'}(F)$. This is the set of molecule types correspond to the terminal set X_n of the sequence

$$F = X_0 \subset X_1, \subset \cdots \subset X_n$$

where X_{i+1} is obtained from X_i by adding each molecule type that is not already in X_i and which can be generated by at least one reaction in R' that has all its reactants in X_i. The set $cl_{R'}(F)$ can be calculated quickly (i.e. in polynomial time in the size of the system (43)).

The graph $\mathcal{G}(R'\ )$

Next, consider the directed graph $\mathcal{G}(R')$ that has vertex set R' and with (r, r') being an arc provided that both of the following condition holds:

- At least one reactant x of r' is a product of r and $x \notin cl_{R' \setminus \{r\}}(F)$.

The following lemma is from Steel *et al.* (119) (Lemma 3.1 and Theorem 1).

Lemma 1. *In any reaction system with a food set (X, R, F) the following four conditions (for any subset R' of R) are equivalent.*

- *R' has an admissible ordering (i.e. R' is F-generated);*
- *$\rho(r) \subseteq cl_{R'}(F)$ for all $r \in R'$;*
- *$cl_{R'}(F) = F \cup \pi(R')$, where $\pi(R') = \cup_{r \in R'} \pi(r)$ (i.e. the set of molecule types generated by the reactions in R').*
- *for each $r \in R', \rho(r) \subseteq \pi(R') \cup F$ and the graph $\mathcal{G}(R')$ has no directed cycles (including any loop on a vertex).*

Next, consider a reaction system with food set, (X, R, F), and a F-generated subset R' of R. It is possible that a given reaction in R' must always occur before (one or several) other given reactions in R' over all admissible ordering of the reaction set. We describe a mathematical result that allows this ordering information to be determined by a polynomial-time algorithm, and represented graphically.

An ordering on R'

We define an order on R' by writing $r \prec r'$ if in every admissible ordering of R' reaction r occurs before r'.

Example 1.1: Consider the following reaction system (which is F-generated):

$$r_1: f_1 + f_2 \rightarrow x;$$

$$r_2: f_2 + f_3 \rightarrow y;$$

$$r_3: f_4 + x \rightarrow z,$$

where $X = \{f_1, f_2, f_3, f_4, x, y, z\}$ and $F = \{f_1, f_2, f_3, f_4\}$. Then, $r_1 \prec r_3$.

To determine whether or not $r \prec r'$, let ξ be a new element that is not present in X. Given a reaction r, let ${}_{+\xi}r$ be the reaction obtained from r by adding ξ as an additional reactant of r. Similarly, given a reaction r', let $r'_{+\xi}$ be the reaction obtained from r' by adding ξ as an additional product of r'. Let $R'[{}_{+\xi}r, r'_{+\xi}]$ be the resulting set of reactions obtained from R' by replacing r with ${}_{+\xi}r$ and r' with $r'_{+\xi}$, respectively.

Proposition 1. *Suppose that R' is an F-generated set, and consider any two distinct reactions r, r' in R'. The following are then equivalent:*

- *$r \prec r'$.*

- $R'[{}_{+\xi}r, r'_{+\xi}]$ *is not* F*-generated.*
- *There is a directed path in* $\mathcal{G}(R')$ *from* r *to* r'.

Proof:

(i) ⇒ (ii): [We show that the negation of (ii) implies (i)] Suppose that the set $R'[{}_{+\xi}r, r'_{+\xi}]$ is F-generated and let o' be an admissible ordering of this set of reactions. Then $r'_{+\xi}$ comes before ${}_{+\xi}r$ in o' (since $r'_{+\xi}$ is the only reaction that produces the reactant ξ required for ${}_{+\xi}r$). Moreover, the ordering obtained from o' by replacing ${}_{+\xi}r$ and $r'_{+\xi}$ with r and r' respectively is an admissible ordering for R' in which r' comes before r and so $r \prec r'$ does not hold.

(ii) ⇒ (iii): Suppose that $R'[{}_{+\xi}r, r'_{+\xi}]$ is not F-generated. Then $R'[{}_{+\xi}r, r'_{+\xi}]$ violates one of the two conditions stated in Part (iv) of Lemma *1*. Since the first condition in Part (iv) clearly holds for this set of reactions, the second must fail - that is $\mathcal{G}(R'[{}_{+\xi}r, r'_{+\xi}])$ contains a directed cycle. However, R' is F-generated, and so (again by Lemma *1*) the graph $\mathcal{G}(R')$ has no directed cycle. Moreover, identifying the vertices of these two graphs (i.e. where r is associated with ${}_{+\xi}r$, and r' with $r'_{+\xi}$) we see that the only difference between the arc sets of these graphs is that $\mathcal{G}(R'[{}_{+\xi}r, r'_{+\xi}])$ contains an arc from $r'_{+\xi}$ to ${}_{+\xi}r$, while $\mathcal{G}(R')$ need not have an arc from r' to r. Since the former graph has a directed cycle but the latter does not, this means that there is a directed path in $\mathcal{G}(R')$ from r to r', as claimed.

(iii) ⇒ (i): Suppose that (r_i, r_j) is an arc in $\mathcal{G}(R')$. Then in any admissible ordering for $\mathcal{G}(R')$ reaction r_i comes before r_j. There is thus a reactant x of r_j that is a product of r_i and, in addition, x is not in the set $cl_{R'\setminus\{r_i\}}(F)$. Thus, in any admissible ordering for R', r_i must come before r_j. Therefore, by transitivity, if there is a directed path from r to r' in $\mathcal{G}(R')$, then r comes before r' in any admissible ordering of R', and therefore $r \prec r'$.

Remark: The ordering $\prec$ can be extended to include any bidirectional reaction(s) r, which has an associated forward reaction r^+ and a backward reaction r^-. In that case, we write $r_1 \prec r_2$ if $r_1^\alpha \prec r_2^\beta$ for all $\alpha, \beta \in \{+, -\}$.

Stratifying the reactions and elements in an F*-generated set*

Given a reaction system with food set (X, R, F) where R is F-generated there is a natural way to partition R into 'levels'. Let $R_0 = \{r \in R: \rho(r) \subseteq F\}$, and for $i \geq 1$, let:

$$R_i = \left\{r \in R: \rho(r) \subseteq F \cup \pi\left(\bigcup_{0\leq j<i} R_j\right)\right\}.$$

By definition, these sets are 'nested' (i.e. $R_i \subseteq R_{i+1}$ for all i). Since R is F-generated, it follows that $R_i = R$ for some value of i (to see this just consider an admissible ordering of R).

For each $r \in R$, we can then define an associated ranking $\lambda(r)$ to be the smallest value of j for which $r \in R_j$. Thus, the collection of reactions in R with values $\lambda(r) = 0,1,2$ ... partitions R into disjoint subsets. Suppose that R is an F-generated set for (X, R, F), and $r, r' \in R$. Then:

$$r \prec r' \Rightarrow \lambda(r) < \lambda(r').$$

To justify this claim, we establish the contrapositive. Suppose that $\lambda(r') \leq \lambda(r)$. Then there is an admissible ordering of R in which r' comes before r. Thus, not every admissible ordering of R' has r before r', so $r \prec r'$ does not hold.

Note also that since R is F-generated and we write $r < r'$ if $r \neq r$ and at least one reactant of r' is a product of r but is not a product of any other reaction in R (e.g., in Example 1.1 we have $r_1 < r_3$), then the condition $r < r'$ implies that $r \prec r'$.

In a similar manner, we can also obtain a stratification of $\Pi(R)$ for any F-generated set R, as follows. Let $X_0 = F$, and for each $i \geq 1$, let:

$$X_i = X_{i-1} \cup \left(\bigcup_{r \in R: \rho(r) \subseteq X_{i-1}} \pi(r) \right).$$

Thus X_i is X_{i-1} together with all the products of all the reactions in R that have all their reactants in X_{i-1}. The resulting collection of sets of elements from X provides a nested increasing sequence of sets $X_0 \subset \cdots \subset X_K = X_{K+1}$, where $X_K = cl_R(F)$. Next, define $\lambda: \pi(R) \rightarrow \{1,2, \ldots\}$ as follows:

$$\lambda(x) = \min\{i: x \in X_i\}.$$

In this way, the elements of $\pi(R)$ are partitioned by their λ values. Moreover, the function λ has the following interpretation: $\lambda(x)$ is the length of the shortest admissible ordering of reactions (from R) that ends in a reaction that has x as a product. Thus if $\lambda(x) < \lambda(y)$, the element x can arise in fewer admissible 'steps' from the food set F than element y can.

The stratification of reactions and molecule types has been implemented into the open-source software package *CatReNet* (118) for use in this paper.

Structures and taxonomic distribution of complexes outside the biosynthetic core

Gene distributions

Protein families obtained as described in **Materials and Methods** were not annotated with sequences for energy conservation enzymes, as they are not part of the autotrophic biosynthetic core. In order to obtain the taxonomic distributions of the Mtr and the Rnf complex (**Fig. S3, Table S6**), one query sequence for each subunit of the complexes was retrieved (GenBank: AJ132817.2 (121) and GenBank: FJ416148.1 (122), respectively). For ATP synthase subunits, all SwissProt prokaryotic sequences that could be identified by a combination of the gene name (**Table S6**) and "ATP synthase" or "ATPase" in the protein name, were retrieved. These sequences were used as queries in DIAMOND blastp alignments against the balanced dataset of 401 archaeal and 552 bacterial genomes (see **Materials and Methods**). BLAST hits with a local sequence identity of ≥25% and an e-value of $\leq 10^{-10}$ were considered to be homologs.

The taxonomic distribution of homologs for each subunit of Mtr and Rnf is shown in **Table S6 and Fig. S3**. The taxonomic distribution of subunits for bacterial (F-type, F-ATPase) and archaeal-type (V-type or V-ATPase) ATP synthases is shown in **Fig. S3**. Universal and domain-specific ATPase subunits were identified based on the literature (123). When BLAST searches were not sensitive enough to detect homologies known from the structural literature, rows were manually joined to reflect known evolutionary relationships (**Table S6**).

Protein structure visualization

Figures of protein structures were rendered with Pymol (The PyMol Molecular Graphics System, Version 2.5.4., Schrödinger, LLC). For ATP synthases (PDB ID: 6OQR (124); PDB ID: 6QUM (125)) domain-specific components were colored according to (114–116, 123, 126). The Rnf structure (PDB ID: 7ZC6 (127)) is missing the small mobile FdI domain. The Mtr structure (PDB ID: 8Q3V (128)) is missing subunits MtrH and $MtrA_s$. They were added for **Fig. S3** similarly as in (128). The complex $Mtr(A_cBGF)_3H_2$ modelled with AlphaFold 3 (36) was superimposed with the experimental structure. MtrH was repositioned to reflect the orientation in (128), and superimposed with the $MtrA_sH_2$ complex modelled with AlphaFold 3 to position $MtrA_s$. The cobalamin cofactor was modelled from a superimposed experimental structure of MtrA with cobalamin (PDB ID: 5LAA) (129). Archaeal transmembrane complexes were embedded in a glycerol-dialkyl-glycerol tetraether (GDGT) (126) lipid monolayer, while bacterial transmembrane complexes were embedded in a palmitoyl-oleoyl-phosphatidylethanolamine (POPE) (130) lipid bilayer. Membrane positioning is schematic.

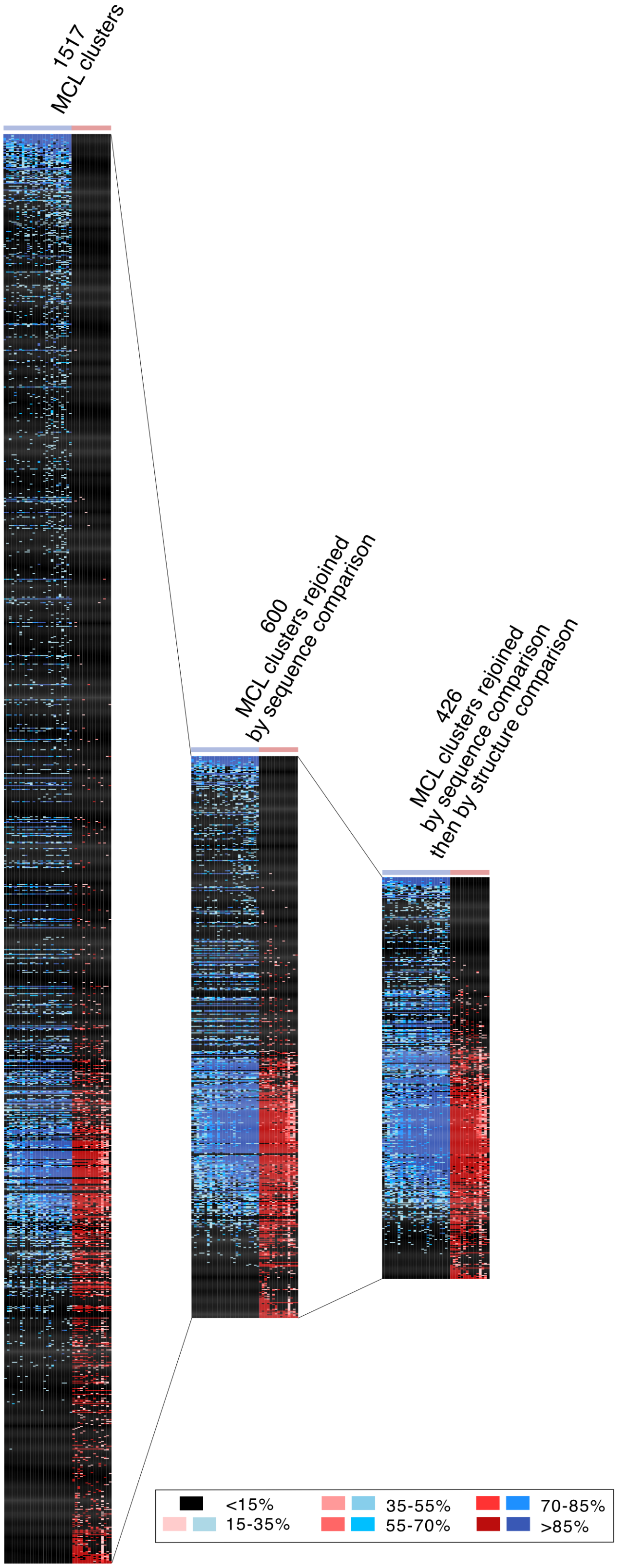

Fig. S1. Merging of core metabolism protein families based on sequence and structural similarity. The distribution of protein families across bacterial classes (blue ticks) and archaeal orders (red ticks) is shown for raw sequence-based MCL clusters (left), clusters after sequence

similarity joining (center) and clusters after subsequent structure similarity joining (right) (**Table S10**). Only clusters that mapped to the same KEGG reaction were checked against joining criteria (**Materials and Methods**). Color intensity indicates the proportion of genomes per taxon possessing the gene (scale at the bottom). Taxa order is the same as in **Fig. 1**.

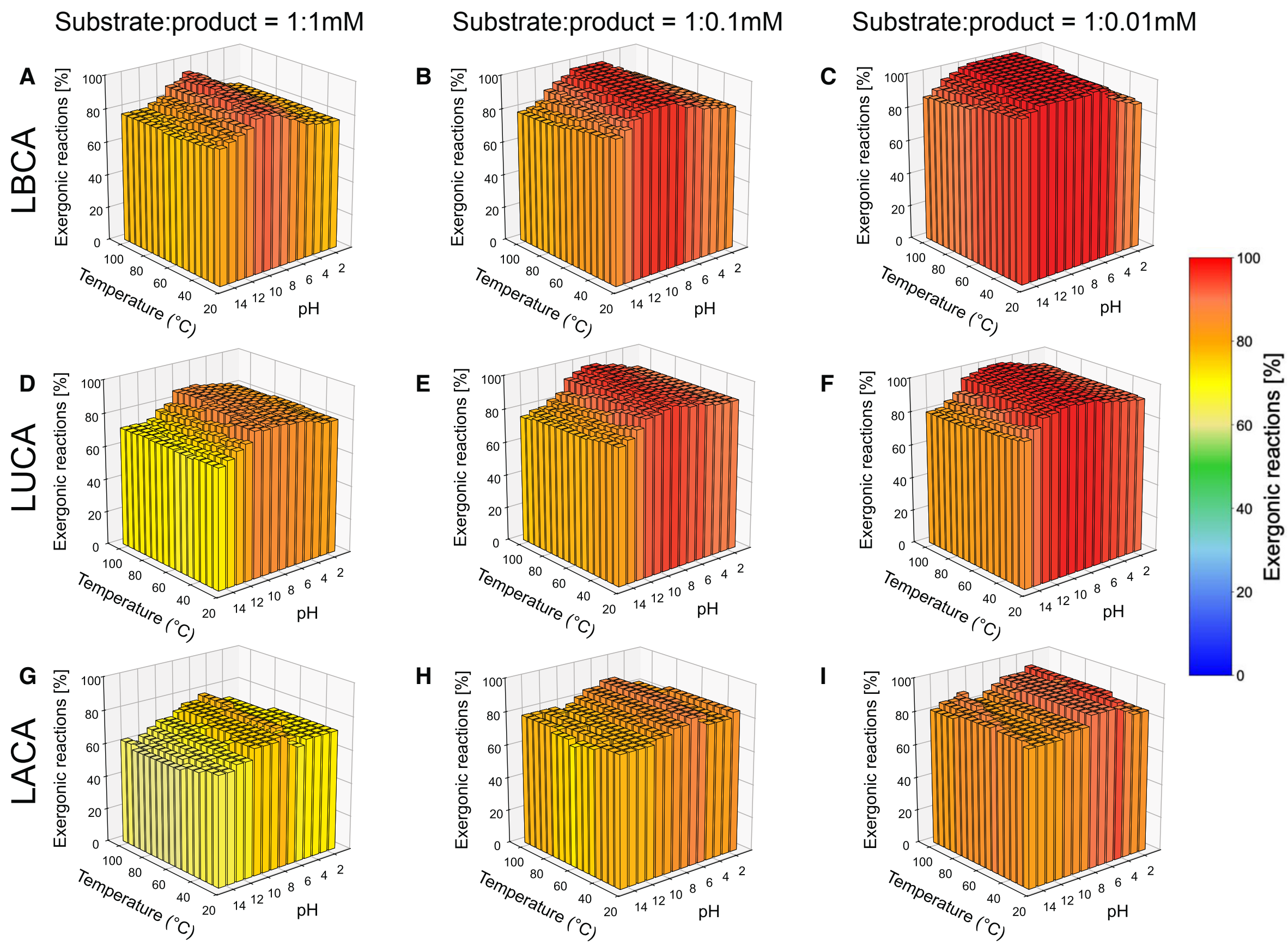

Fig. S2. Proportions of exergonic reactions in LBCA, LUCA and LACA. Proportions of exergonic and close-to-equilibrium reactions (ΔG < 5 kJ/mol) are plotted for each set of conditions for 326 reactions that yielded a ΔG value. Ionic strength (250 mM) and reactant concentration (1 mM) are constant, while product concentration is decreased from 1mM (**A**, **D**, **G**) to 0.1 mM (**B**, **E**, **H**) and 0.01 mM (**C**, **F**, **I**). Plots for reactions assigned to LBCA are shown in **A**, **B** and **C**, plots for reactions assigned to LUCA are in **D**, **E** and **F**, while reactions assigned to LACA are shown in **G**, **H** and **I**. Grey zones from **Fig. 1B** were excluded. Underlying data can be found in **Table S5**.

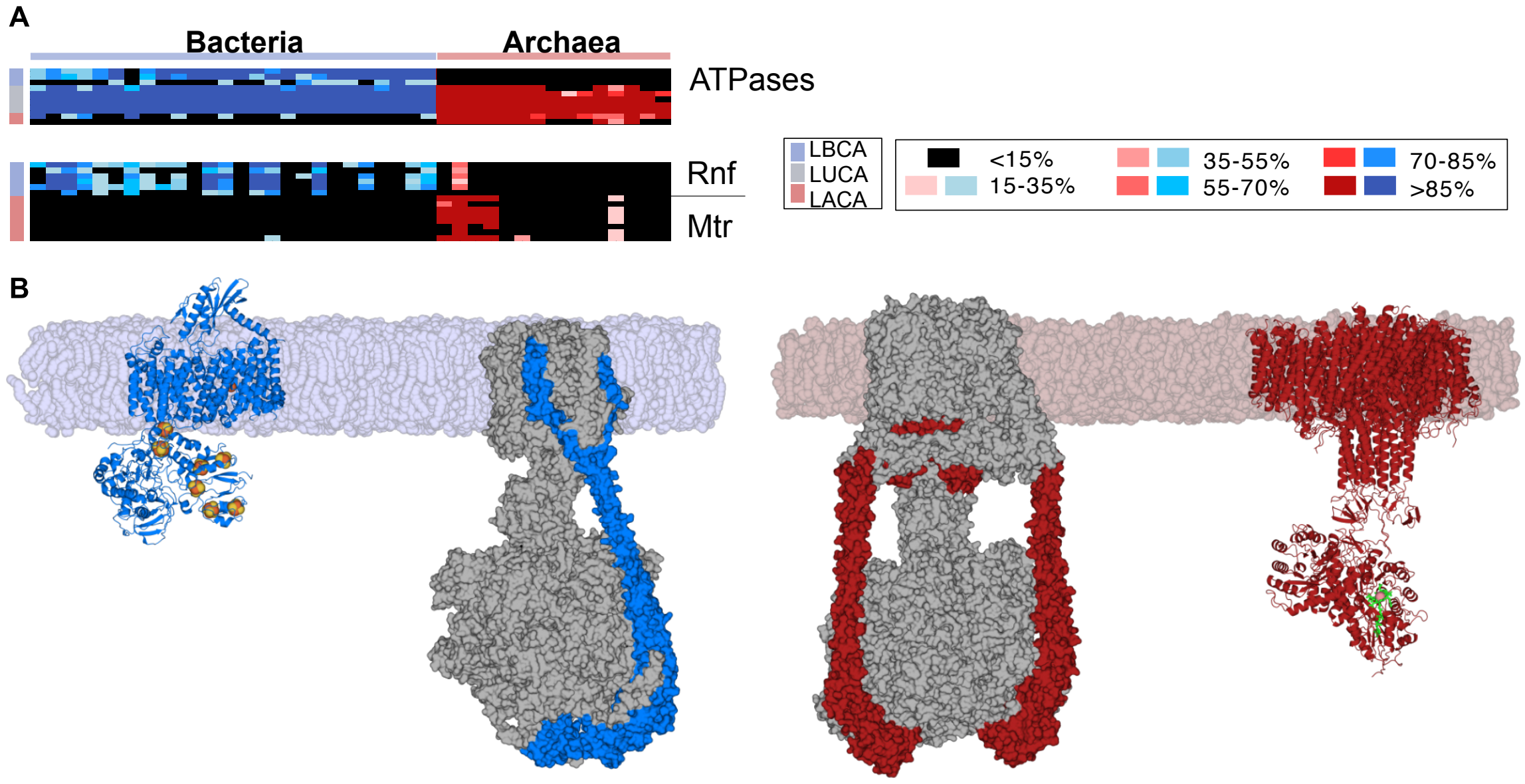

Fig. S3. Distribution and protein structures of ancestral energy conservation enzymes in bacteria and archaea. For escape as free-living cells, metabolism had to achieve the state of a balanced stoichiometric reaction (131), called redox balance in physiology, and the exergonic reactions of electron transfer from H_2 to CO_2 needed to be coupled to the pumping of ions from the inside of the cell to the outside (chemiosmotic coupling), as in modern cells. Prior to the coupling of CO_2 reduction to ion pumping, the natural pH gradient provided by serpentinizing systems (pH 9–11, vs. sea water (pH 6.5 (132)) was harnessable with the help of simple, abiotically synthesized hydrophobics (133, 134) such as fatty acids, which were recently shown to support the ATP-synthesizing function of a rotor-stator ATPase (135). **A**. The distribution pattern of genes for ATPase subunits resembles ribosomal proteins (**Fig. 1A**) and core metabolism (**Fig. 1B**). The ATPase needed to harness geochemically generated ion gradients is an invention of the protein world and has no geochemical analogue. In order to replace the natural pH gradient by a biological mechanism, much like replacement of solid-state metals by cofactors, enzyme innovations were required. Gradient-generating pumping complexes originated separately in the bacterial (Rnf) and archaeal (Mtr) domains. The occurrence of Rnf in Methanosarcinales, the most recent order of methanogens (54), is known and likely due to lateral gene transfer from bacteria (136). Genes present in bacterial genomes are plotted as blue ticks, while those in archaeal genomes are in red. Color intensity indicates the proportion of genomes per taxon possessing the gene, according to the scale in **Fig. 1**. For details see **Table S6**. **B**. Structures of the ATP synthase (124, 125), Rnf (127) and Mtr (36, 128, 129) are shown embedded in bacterial and archaeal lipids. Before the independent origin of lipid synthesis in bacteria and archaea, the ATP synthase could have functioned in simple abiotically synthesized

fatty acid membranes (135). ATP synthase subunits that arose independently in the two domains are shown in red (archaea) and blue (bacteria) (123). The cobalamin cofactor in Mtr is shown in green. FeS clusters are highlighted.

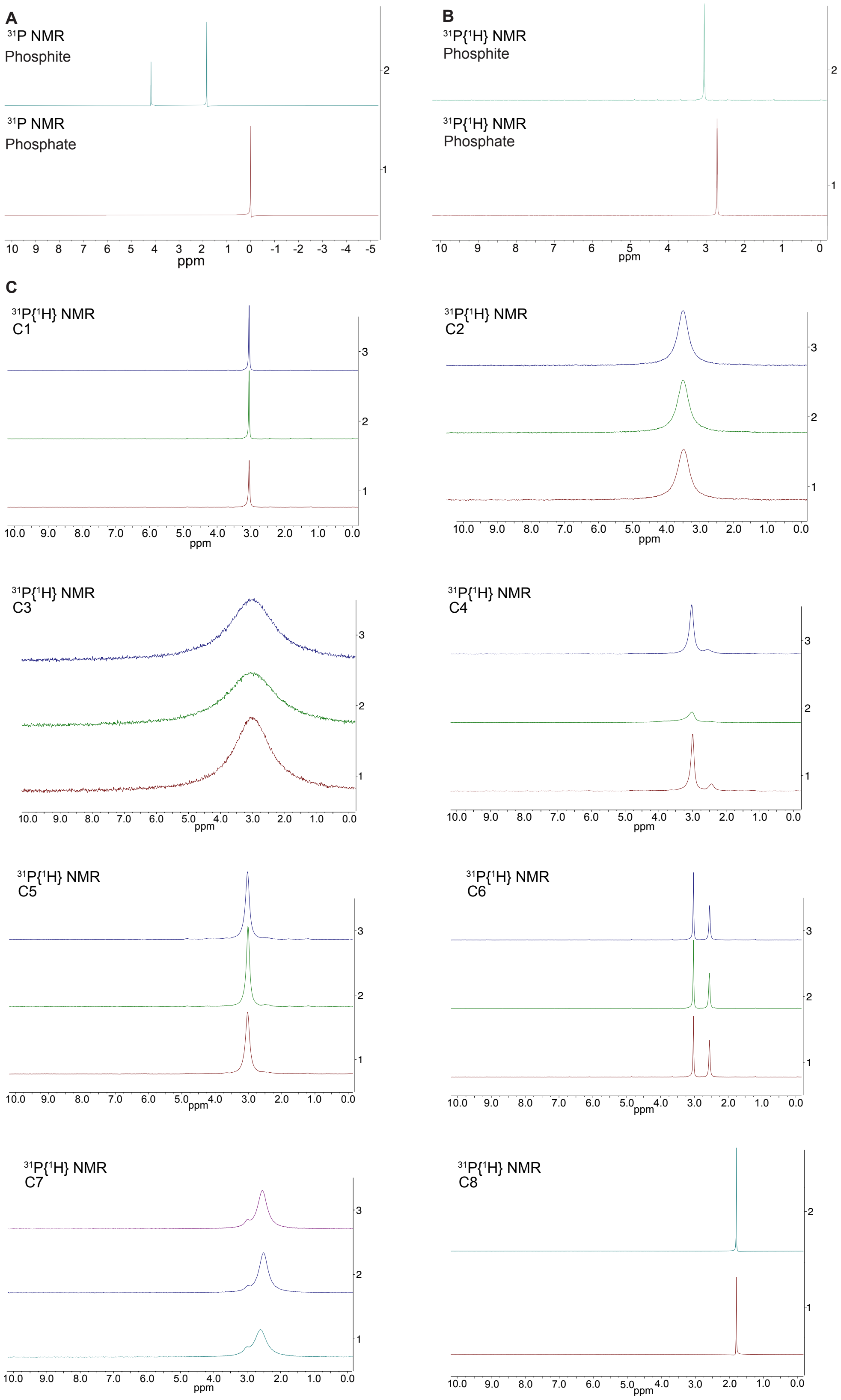
A
^{31}P NMR
Phosphite
^{31}P NMR
Phosphate
B
^{31}P{^{1}H} NMR
Phosphite
^{31}P{^{1}H} NMR
Phosphate
ppm
C
^{31}P{^{1}H} NMR
C1
^{31}P{^{1}H} NMR
C2
^{31}P{^{1}H} NMR
C3
^{31}P{^{1}H} NMR
C4
^{31}P{^{1}H} NMR
C5
^{31}P{^{1}H} NMR
C6
^{31}P{^{1}H} NMR
C7
^{31}P{^{1}H} NMR
C8
ppm

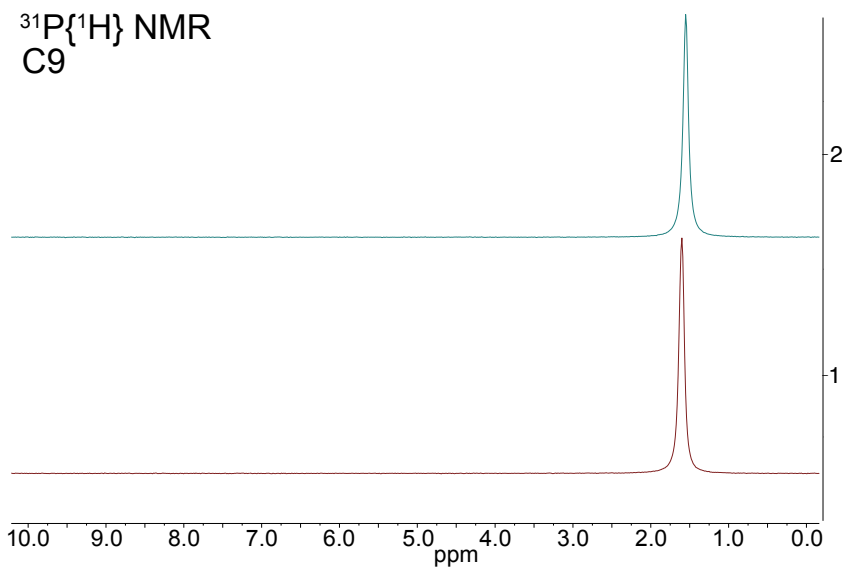

D

Phosphite	Y = 2654577*X					
Number	C1 - 1	C1 - 2	C1 - 3	C2 - 1	C2 - 2	C2 - 3
Peak area	504904842.8	537129478.3	501256867.1	515612407.6	520742115.9	482383474.6
Range	3.30 .. 2.79	3.45 .. 2.88	3.48 .. 2.72	6.50 .. 1.08	6.84 .. 0.78	5.08 .. 2.04
[%] Reactant	97.08			99.21		
Number	C3 - 1	C3 - 2	C3 - 3	C4 - 1	C4 - 2	C4 - 3
Peak area	523528584.1	501287103.1	548024242.5	307134782.5	418839145.6	294368170.6
Range	10.34 .. -3.89	11.80 .. -5.02	7.93 .. -2.07	4.32 .. 2.65	5.01 .. 2.78	4.25 .. 2.72
[%] Reactant	100.28			90.85		
Number	C5 - 1	C5 - 2	C5 - 3	C6 - 1	C6 - 2	C6 - 3
Peak area	530068031	514601543.3	524254441.8	282730340.3	294682964.3	251745488
Range	4.23 .. 2.01	4.13 .. 2.08	4.13 .. 1.98	3.61 .. 2.84	3.51 .. 2.80	3.31 .. 2.80
[%] Reactant	99.85			52.76		
Number	C7 - 1	C7 - 2	C7 - 3			
Peak area	40832622	52254441.75	133123559.3			
Range	4.36 .. 3.35	4.79 .. 2.95	4.95 .. 2.96			
[%] Reactant	22.8					

Phosphate	Y = 1363794*X					
Number	C4 - 1	C4 - 2	C4 - 3	C6 - 1	C6 - 2	C6 - 3
Peak area	120172930.9	37382853.5	96519876.62	136877987	152772728.3	143981088.5
Range	2.67 .. 1.37	2.78 .. 1.84	2.72 .. 1.98	2.84 .. 2.06	2.81 .. 2.26	2.79 .. 2.22
Average yield [%]	14.12			55.39		
Number	C7 - 1	C7 - 2	C7 - 3			
Peak area	40832622	52254441.75	133123559.3			
Range	4.36 .. 3.35	4.79 .. 2.95	4.95 .. 2.96			
Average yield [%]	83.16					
Number	C8 - 1	C8 - 2	C9 - 1	C9 - 2		
Peak area	282592437	264533092	273910446.8	285342174.3		
Range	1.40 .. 1.15	1.51 .. 1.11	1.90 .. 1.53	1.86 .. 1.54		
Average yield [%]	100.3		102.52			

E

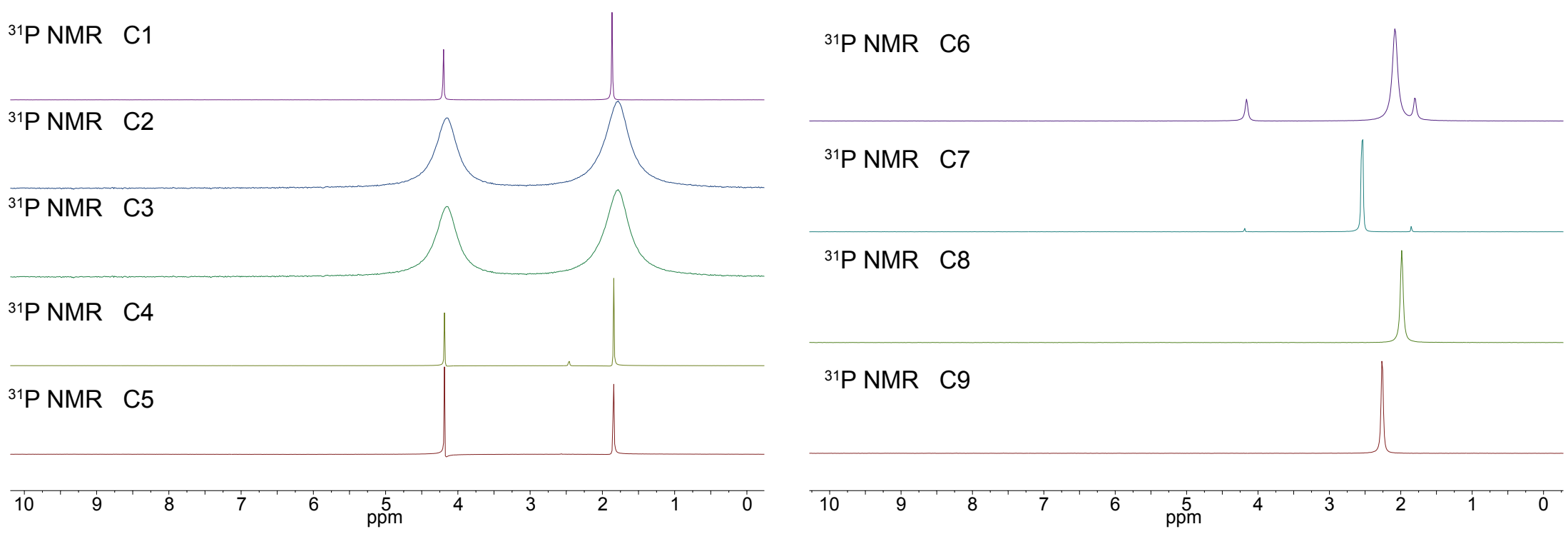

Fig. S4. ^{31}P NMR data for phosphite oxidation reactions. A. Proton-coupled ^{31}P NMR spectra of 200 mM phosphite and 200 mM phosphate standards in water at 100 °C and pH 9, with phosphite displaying the characteristic broad doublet and phosphate appearing as a singlet. **B.** Proton-decoupled ^{31}P NMR spectra of 200 mM phosphite and 200 mM phosphate standards

in water at 100 °C and pH 9. These standards were used to distinguish phosphite from phosphate in the reaction spectra. **C.** Proton-decoupled ^{31}P NMR spectra of phosphite oxidation reactions corresponding to conditions C1–C9 in **Fig. 4A**, shown in the same order as the columns in **Fig. 4A**. Results of three (conditions C1–C7) or two (conditions C8 and C9) replicates are shown. Details on experimental conditions for each reaction are listed in **Table S7**. **D.** Peak areas, integration ranges and average phosphate yield/remaining phosphite (in percentages) for the ^{31}P signals in spectra shown in C (conditions C1–C9). Peak areas were obtained by integration over the indicated chemical shift ranges and converted to concentrations using phosphite and phosphate calibration curves derived from an independent concentration series (calibration curves shown in red). Results are shown as bar plots in **Fig. 4A**. **E.** Proton-coupled ^{31}P NMR spectra corresponding to conditions C1–C9 (for details see **Fig. 4A** and **Table S7**), used to confirm signal assignments. Nagaosa and Aoyama (137) reported efficient phosphite oxidation using 10% Pd on carbon in the presence of O_2, they suggested a mechanism involving an H_2O_2 intermediate. Our reactions were performed under anoxic conditions under Ar. In a standard phosphite to phosphate conversion experiment (400 ml reactor with 10.5 ml total volume of 200 mM phosphite), complete phosphite oxidation should generate 2.1 mmol H_2, according to $H_3PO_3 + H_2O \rightarrow H_3PO_4 + H_2$. In such reactions, the observed pressure increase in the reactor (0.5 L volume) is ~0.5 bar, corresponding to the accumulation of 0.05 L (2.2 mmol) of H_2 gas as a reaction product.

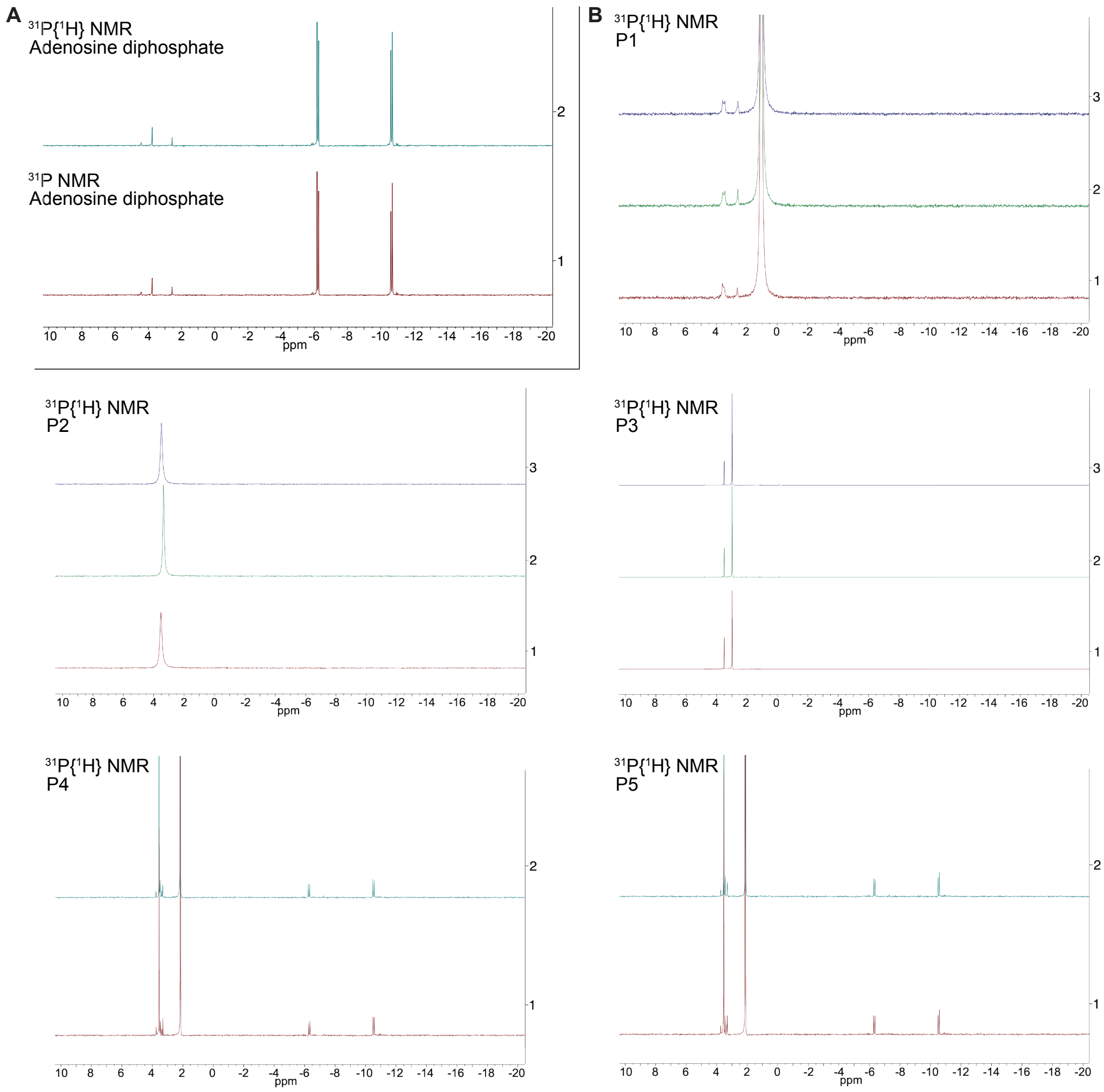

C

ADP	2654272*X - 7219126			
Number	P4 - 1	P4 - 2	P5 - 1	P5 - 2
Peak area	13279062.25	13020027.75	15083206.25	14427535.5
Range	-10.38 .. -10.70	-10.33 .. -10.76	-10.38 .. -10.75	-10.40 .. -10.70
Average yield [%]	7.67		8.28	

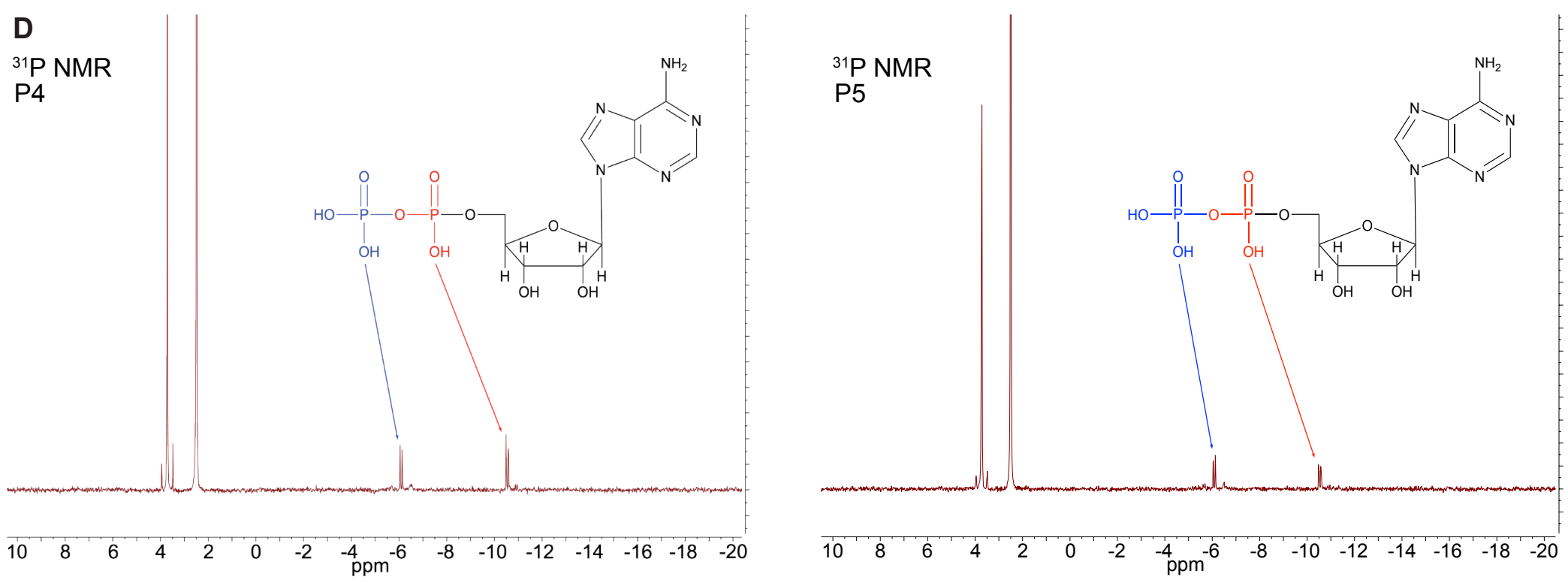

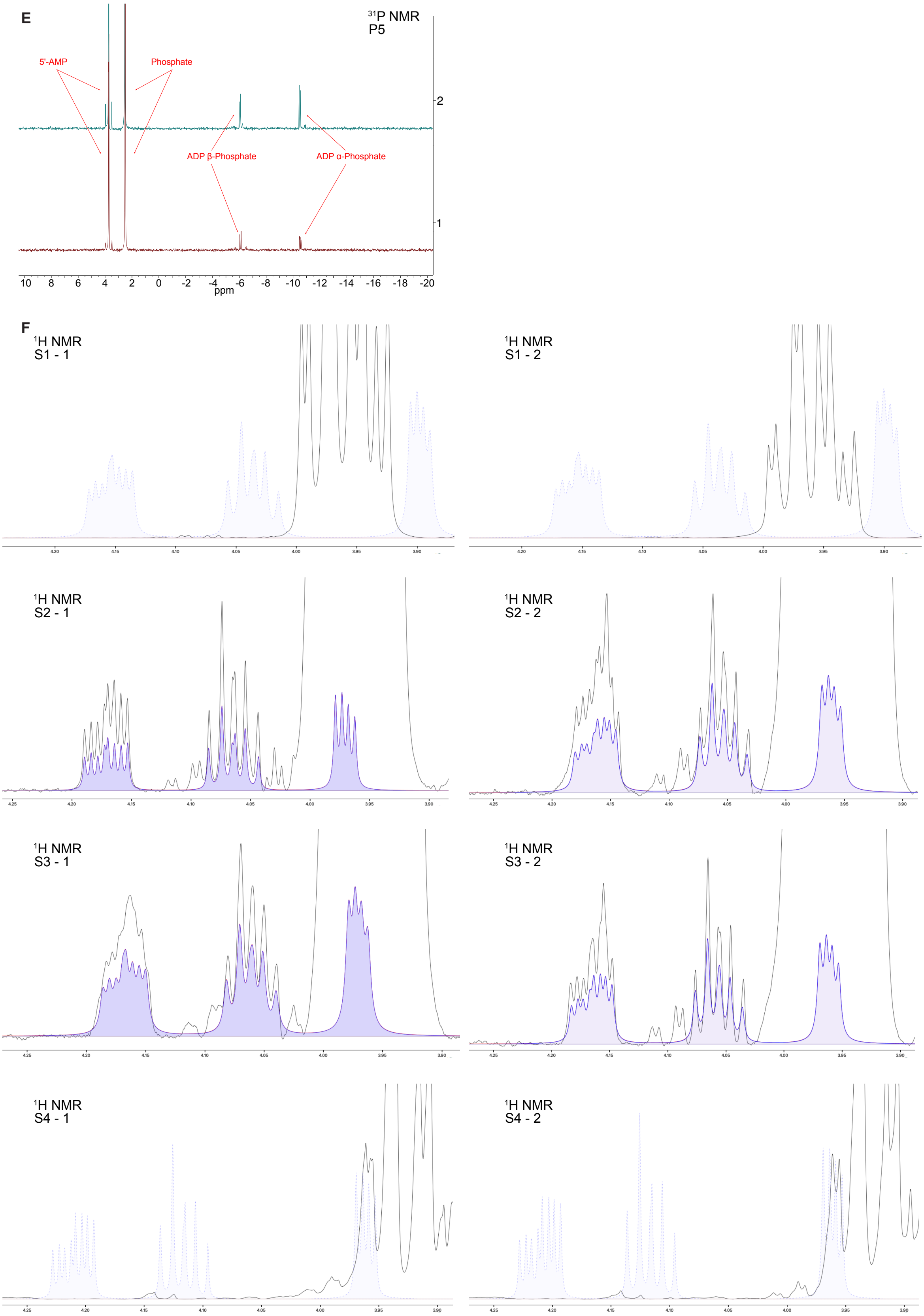
E
^{31}P NMR
P5
5'-AMP
Phosphate
ADP β-Phosphate
ADP α-Phosphate
2
1
10 8 6 4 2 0 -2 -4 -6 -8 -10 -12 -14 -16 -18 -20
ppm
F
^{1}H NMR
S1 - 1
^{1}H NMR
S1 - 2
^{1}H NMR
S2 - 1
^{1}H NMR
S2 - 2
^{1}H NMR
S3 - 1
^{1}H NMR
S3 - 2
^{1}H NMR
S4 - 1
^{1}H NMR
S4 - 2

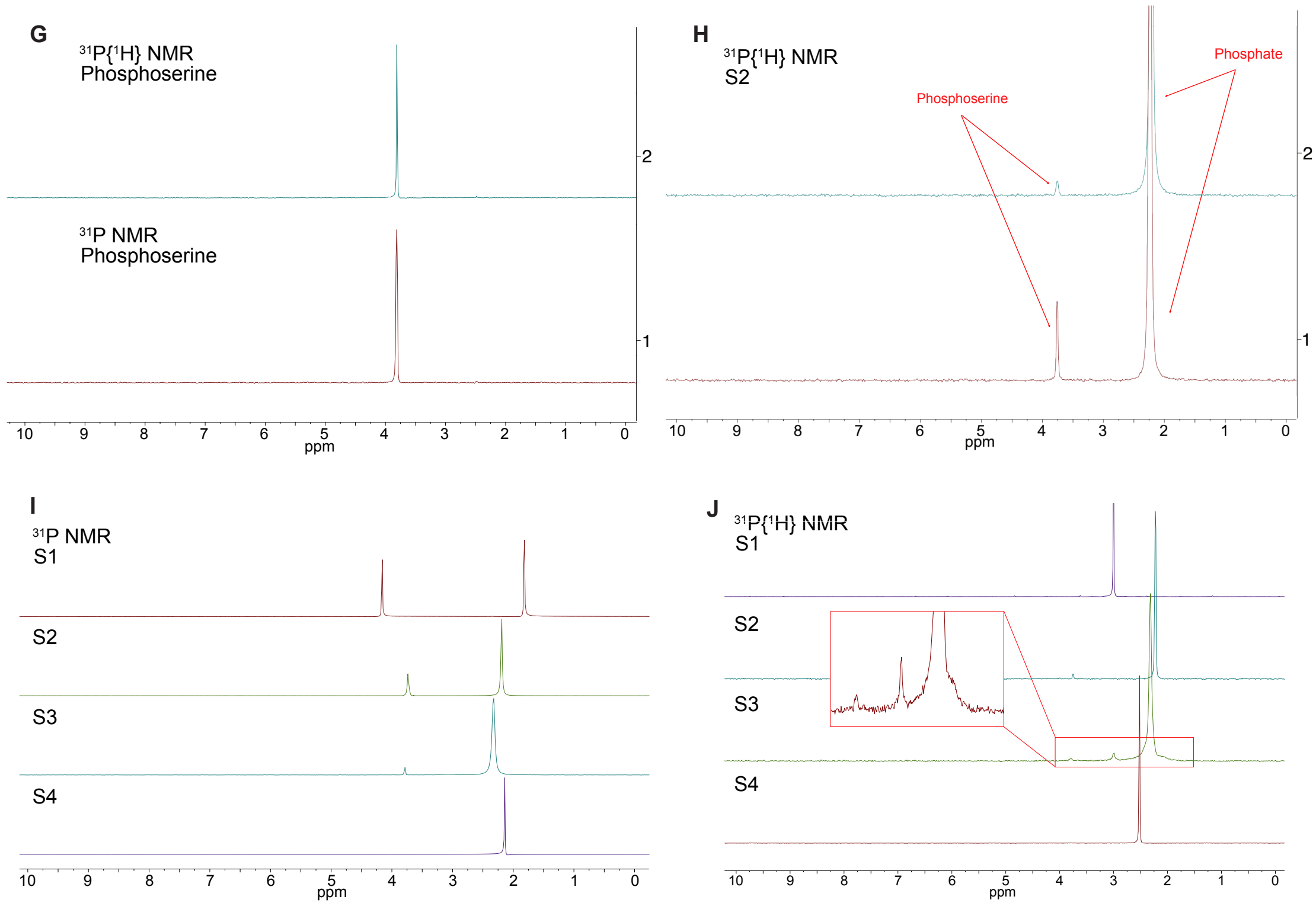

Fig. S5. NMR data for phosphite-dependent phosphorylation of AMP and serine over Pd/C. A. ^{31}P NMR spectra of ADP standards (200 mM) recorded in both proton-coupled (^{31}P NMR) and proton-decoupled ($^{31}P\{^1H\}$ NMR) modes at 50 °C and pH 9. These spectra were used to assign ADP signals in the reaction mixtures. **B.** Proton-decoupled ^{31}P NMR spectra of AMP phosphorylation reactions corresponding to conditions P1–P5 in **Fig. 4B**. These spectra were used to quantify ADP. Results of three (conditions P1–P3) or two (conditions P4 and P5) replicates are shown. Details of experimental conditions are listed in **Table S7**. **C.** Peak areas, integration ranges and product yields for the ^{31}P signals corresponding to ADP in spectra P4–P5. Peak areas were obtained by integration over the indicated chemical shift ranges and converted to concentrations using an ADP calibration curve derived from an independent concentration series (calibration curve shown in red). Results are shown as bar plots in **Fig. 4B**. **D.** Proton-coupled ^{31}P NMR spectra of AMP phosphorylation reactions under conditions P4 and P5, showing the ADP region with assignment of the α- and β-phosphate signals. **E.** Proton-coupled ^{31}P NMR spectra of the AMP phosphorylation reaction under condition P5 before (bottom) and after spiking with 10 mM ADP (top). The selective increase of the α- and β-ADP signals in the spiked spectrum supports their assignment. **F.** ^1H NMR spectra of serine phosphorylation reactions corresponding to conditions S1–S4 in **Fig. 4C**. Results of two replicates are shown next to each other for each experimental condition (S1–S4). Yields are shown as bar plots in **Fig. 4C**. Blue spectra represent phosphoserine standards (from the internal

Chenomx software library), while black spectra represent reaction samples (overlaid). The top of the spectra has been truncated for reasons of space. Dashed blue lines indicate the absence of phosphoserine. **G.** ^{31}P NMR spectra of phosphoserine standards recorded in both proton-coupled (^{31}P NMR) and proton-decoupled (^{31}P{^{1}H} NMR) modes at 50 °C and pH 9. **H.** Proton-decoupled ^{31}P NMR spectrum of the serine phosphorylation reaction under condition S2. The top spectrum shows the reaction mixture, and the bottom spectrum shows the same sample after spiking with phosphoserine, confirming the assignment of the phosphoserine signal. **I.** Proton-coupled ^{31}P NMR spectra of serine phosphorylation reactions corresponding to conditions S1–S4. **J.** Proton-decoupled ^{31}P{^{1}H} NMR spectra of serine phosphorylation reactions corresponding to conditions S1–S4. All reaction conditions are described in **Materials and Methods** and **Table S7**.

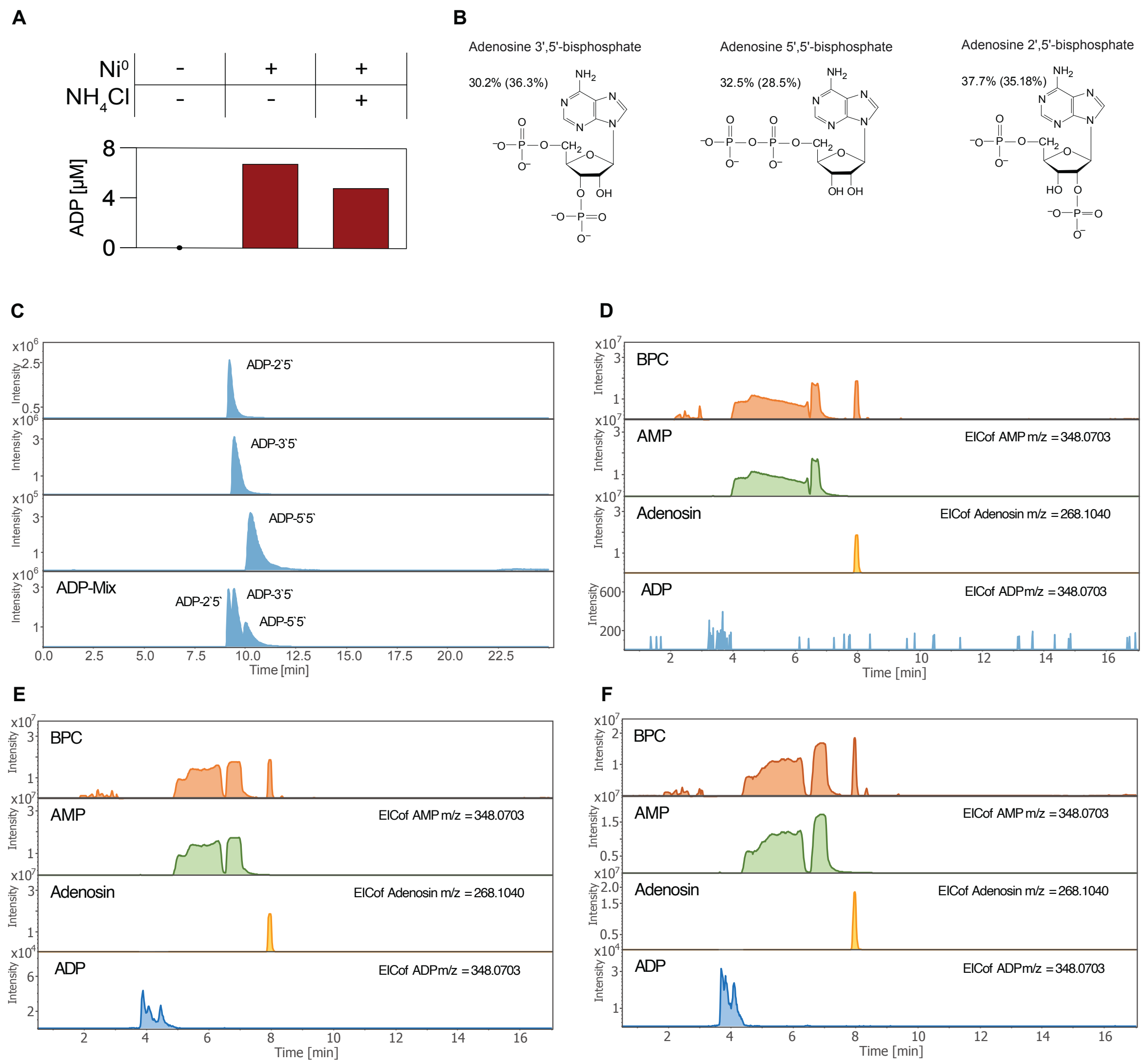

Fig. S6. ESI-LC-MS data for phosphite-dependent phosphorylation of AMP to ADP over Ni^0. A. Concentration of ADP formed from AMP in the presence or absence of NH_4Cl and Ni^0 in a 96 h reaction at pH 7 and 50 °C. Values are derived from ESI-LC-MS measurements (spectra shown in panels D, E and F). **B.** Structures of the three ADP isomers. Percentage values outside parentheses indicate the proportions of ADP isomers in the absence of NH_4Cl, while values in parentheses refer to reactions conducted with NH_4Cl; the proportions are calculated from the data in panels D, E, and F. **C.** ESI-LC-MS standards of the three ADP isomers and their mixture. **D.** ESI-LC-MS analysis of a reaction mixture without Ni^0 and without NH_4Cl (first condition in panel A). **E.** ESI-LC-MS analysis of a reaction mixture with Ni^0 and without NH_4Cl (second condition in panel A). **F.** ESI-LC-MS analysis of a reaction mixture with Ni^0 and NH_4Cl (last condition in panel A).

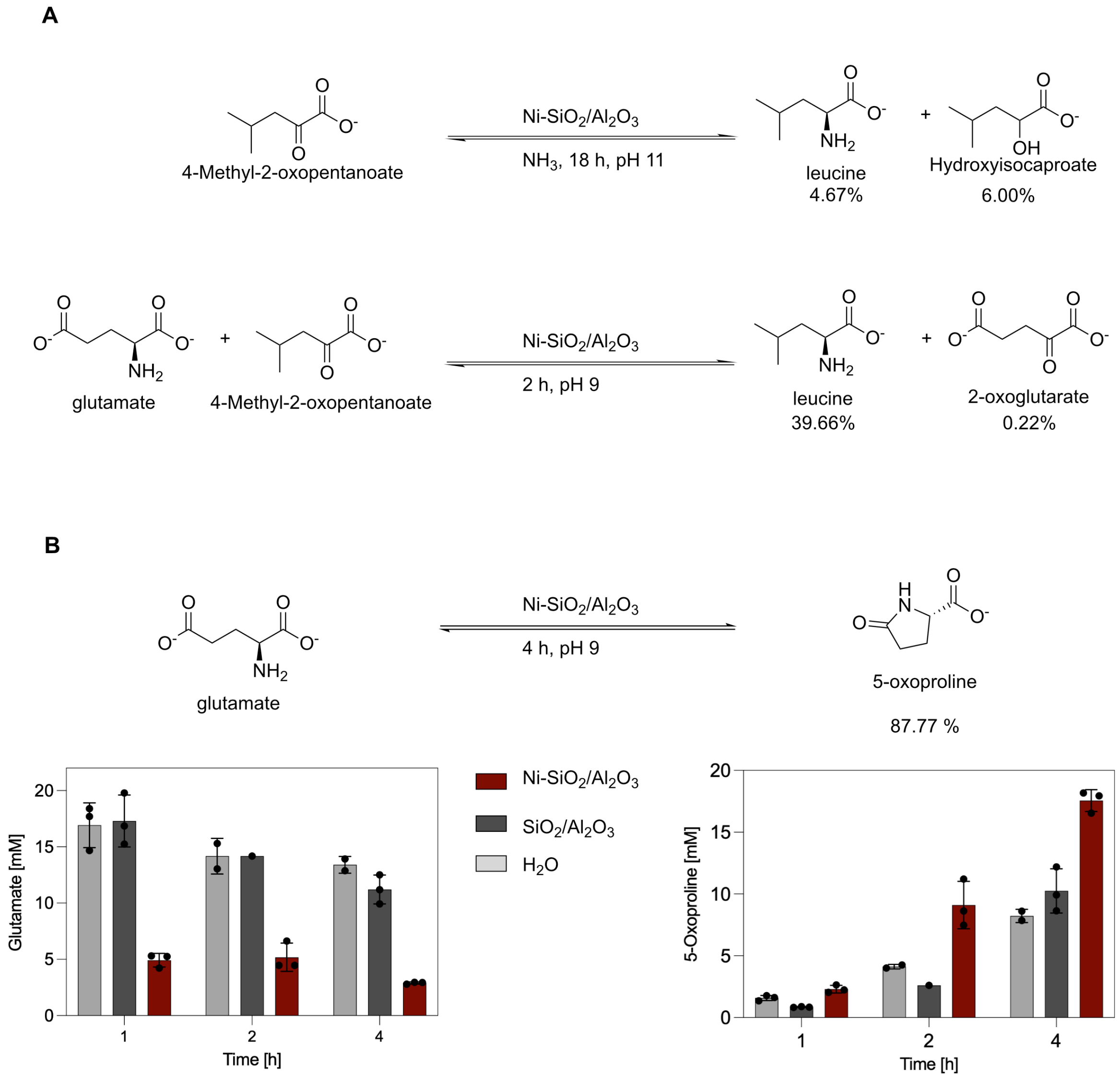

Fig. S7. Reactions experimentally shown in this work under simulated hydrothermal conditions. **A.** Core metabolism reactions with their associated KEGG identifiers. **B.** Intramolecular cyclization of glutamate into 2-oxoproline (pyroglutamate) with amide bond formation. Concentrations of the reactant (glutamate) and product (2-oxoproline) are plotted after a reaction time of 1 h, 2 h, and 4 h with different catalysts. Reaction conditions are described in **Materials and Methods**, with specific conditions marked underneath the arrows. Yields are shown underneath each product.

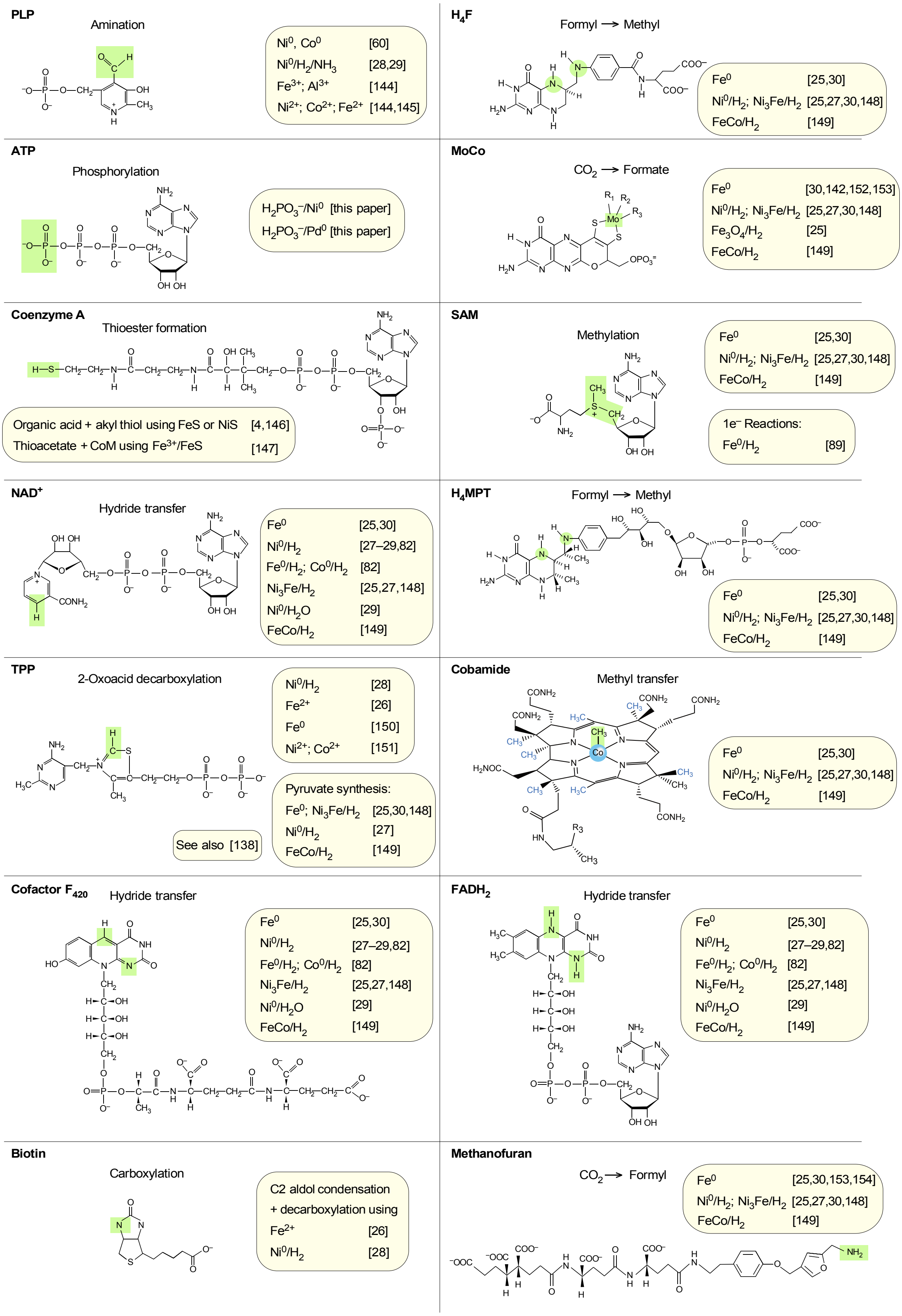

Fig. S8. Functions of cofactors that are replaced by metals. Metals that catalyze the reaction are indicated in shaded boxes. References are indicated in brackets. PLP can perform a number

of functions performed by TPP (138) and is likely the evolutionary precursor of TPP (138). Water is the most common reactant in metabolism, indicating that metabolism arose in the aqueous phase, but studies employing gas phase reactions achieve CO_2 fixation using H_2 and Co^0, Fe^0, and alloys (139) or ultraviolet-light enhanced CO_2 fixation over metal sulfide catalysts (140). For the conversions cited in the figure, catalysts are essential. In the absence of catalysts, CO_2 conversion to formate or methane is negligible, even after 5 years at 300°C and 350 bar (141), whereas in the presence of native metal catalysis, 0.1–0.5 M formate can be produced in water from H_2 and CO_2 gas overnight (25, 27, 30) (reviewed (142)). Ion gradients are, however, not required for any of the reactions shown here. It has been reported that an ion gradient can convert CO_2 to 1 μM formate (143), yet 200,000 times higher formate concentrations (in addition to C2 and C3 products) are obtained from H_2 and CO_2 using of Ni^0 or Ni_3Fe (25, 27, 30).

Table S1. (separate file) Distribution of ribosomal proteins across prokaryotic higher taxa.

The file contains the distribution of 83 ribosomal proteins across bacterial classes and archaeal orders and a list of ribosomal proteins from *E. coli* and *T. kodakarensis* used to retrieve ribosomal protein sequences. Universal and domain-specific ribosomal proteins are marked.

Table S2. (separate file) Distribution of core metabolic enzymes across prokaryotic higher taxa and substrate generality assignment.

The file contains core biosynthetic enzyme distributions across bacterial classes and archaeal orders and their assignment to LUCA, LBCA or LACA. Additionally, the overlap with KOs identified in a recent genome-wide inference of LUCA based on phylogeny from Moody *et al.* (19) and literature references for the characterization of LACA and LBCA enzymes are listed. A sheet with the assignment of substrate generalists (enzymes with broad substrate specificity) in the biosynthetic core is also provided. Substrate generality is traditionally understood as an essential property of primordial enzymes, as it reduces the number of enzymes that the first cells required. The generality of enzymes (accepting many substrates) and cofactors (each used by many enzymes) was preceded in metabolic assembly by substrate generality of metabolism's metallic inorganic precursors.

Table S3. (separate file) Types of chemical reactions catalyzed by core metabolism protein families.

Reaction equations and assignments of reaction types are given for 361 reactions assigned to protein families.

Table S4. (separate file) Core metabolic reactions reported experimentally under serpentinizing vent conditions.

The file contains a referenced list of core metabolic reactions mapped to protein families that have been experimentally shown to proceed under hydrothermal conditions, without catalysis or catalyzed by transition metals. Reactions shown to occur on FeS minerals, Fe(II/III) oxyhydroxides or with the use of an external electric current are marked in a separate column. The reactions shown experimentally are assigned to three categories: A. The reaction has been reported experimentally in hydrothermal conditions; B. The same chemical transformation on a different substrate has been reported experimentally; C. The reaction or pathway it belongs

to has been reported experimentally, even though the pathway from reactants to products in the laboratory involves a different number of steps or a different reaction sequence compared to metabolism.

Table S5. (separate file) Gibbs energy ΔG for core metabolic reactions.
Gibbs energy values (ΔG) of core metabolic reactions are given across a range of pH, temperatures and concentrations. Only values for reactions mapped to protein families that yielded a ΔG value are listed.

Table S6. (separate file) Distribution of ATPase, Mtr and Rnf subunits across prokaryotic higher taxa.
The file contains the distribution of genes for ancient energy conservation complexes (the ATP synthase, Mtr and Rnf) across bacterial classes and archaeal orders.

Table S7. (separate file) Reaction conditions for aqueous phosphite oxidation and phosphite-dependent phosphorylation over metals. Reactant concentrations, added metals, pH, temperature, atmosphere and reaction duration are listed. All reactions were carried out under anoxic conditions in aqueous solution.

Table S8. (separate file) Input data and results of reaction stratification.
The file contains the food set and reaction set used in the stratification analysis, a list of excluded and added reactions, notes on recoding and the output given by the CatReNet software.

Table S9. (separate file) Initial set of 424 core metabolic reactions.
The file contains reaction equations for 424 metabolic reactions required to synthesize amino acids, nucleotides and cofactors from H_2, CO_2, NH_3, P_i, H_2O and H_2S. They are polarized in the biosynthetic direction, except for irreversible glycolytic reactions. Reactions added in this study to the original dataset from Wimmer *et al.* (17) are marked.

Table S10. (separate file) List of protein families after sequence-based and structure-based merging and TM-scores for structural alignments.
The file contains protein families mapped to reaction IDs at each stage of sequence- and structure-based cluster merging. TM-scores obtained for structural alignments are listed.

REFERENCES

1. B. Damer, D. Deamer, The hot spring hypothesis for an origin of life. *Astrobiology* **20**, 429–452 (2020).

2. D. D. Sasselov, J. P. Grotzinger, J. D. Sutherland, The origin of life as a planetary phenomenon. *Sci. Adv.* **6**, eaax3419 (2020).

3. G. Wächtershäuser, Before enzymes and templates: Theory of surface metabolism. *Microbiol. Rev.* **52**, 452–484 (1988).

4. C. Huber, G. Wächtershäuser, Activated acetic acid by carbon fixation on (Fe,Ni)S under primordial conditions. *Science* **276**, 245–248 (1997).

5. C. B. Messner, P. C. Driscoll, G. Piedrafita, M. F. L. De Volder, M. Ralser, Nonenzymatic gluconeogenesis-like formation of fructose 1,6-bisphosphate in ice. *Proc. Natl. Acad. Sci. U.S.A.* **114**, 7403–7407 (2017).

6. J. B. Corliss, J. A. Baross, S. E. Hoffman, An hypothesis concerning the relationship between submarine hot springs and the origin of life on Earth. *Oceanol. Acta* **4**, 59–69 (1981).

7. W. Martin, J. Baross, D. Kelley, M. J. Russell, Hydrothermal vents and the origin of life. *Nat. Rev. Microbiol.* **6**, 805–814 (2008).

8. N. Lane, W. F. Martin, The origin of membrane bioenergetics. *Cell* **151**, 1406–1416 (2012).

9. F. L. Sousa, T. Thiergart, G. Landan, S. Nelson-Sathi, I. A. C. Pereira, J. F. Allen, N. Lane, W. F. Martin, Early bioenergetic evolution. *Philos. Trans. R. Soc. Lond. B Biol. Sci.* **368**, 20130088 (2013).

10. L. E. Orgel, The implausibility of metabolic cycles on the prebiotic Earth. *PLoS Biol.* **6**, e18 (2008).

11. S. A. Kauffman, *The Origins of Order* (Oxford Univ. Press, 1993).

12. M. A. Pasek, A. Omran, T. Feng, M. Gull, C. Lang, J. Abbatiello, L. Garong, R. Johnston, J. Ryan, H. Abbott-Lyon, Serpentinization as a route to liberating phosphorus on habitable worlds. *Geochim. Cosmochim. Acta* **336**, 332–340 (2022).

13. W. Buckel, Anorganische Chemie in Meeressedimenten. *Angew. Chem.* **113**, 1463–1464 (2001).

14. A. Kirschning, Coenzymes and their role in the evolution of life. *Angew. Chem. Int. Ed. Engl.* **60**, 6242–6269 (2021).

15. J. D. Sutherland, Opinion: Studies on the origin of life — The end of the beginning. *Nat. Rev. Chem.* **1**, 0012 (2017).

16. A. do Nascimento Vieira, K. Kleinermanns, W. F. Martin, M. Preiner, The ambivalent role of water at the origins of life. *FEBS Lett.* **594**, 2717–2733 (2020).

17. J. L. E. Wimmer, J. C. Xavier, A. do Nascimento Vieira, D. P. Henriques Pereira, J. Leidner, F. L. Sousa, K. Kleinermanns, M. Preiner, W. F. Martin, Energy at origins: Favorable thermodynamics of biosynthetic reactions in the last universal common ancestor (LUCA). *Front. Microbiol.* **12**, 793664 (2021).

18. M. C. Weiss, F. L. Sousa, N. Mrnjavac, S. Neukirchen, M. Roettger, S. Nelson-Sathi, W. F. Martin, The physiology and habitat of the last universal common ancestor. *Nat. Microbiol.* **1**, 16116 (2016).

19. E. R. R. Moody, S. Álvarez-Carretero, T. A. Mahendrarajah, J. W. Clark, H. C. Betts, N. Dombrowski, L. L. Szánthó, R. A. Boyle, S. Daines, X. Chen, N. Lane, Z. Yang, G. A. Shields, G. J. Szöllősi, A. Spang, D. Pisani, T. A. Williams, T. M. Lenton, P. C. J. Donoghue, The nature of the last universal common ancestor and its impact on the early Earth system. *Nat. Ecol. Evol.* **8**, 1654–1666 (2024).

20. A. Kornberg, "On the metabolic significance of phosphorolytic and pyrophosphorolytic reactions," in *Horizons in Biochemistry*, H. Kasha, B. Pullman, Eds. (Academic Press Inc., 1962), pp. 251–264.

21. K. Decker, K. Jungermann, R. K. Thauer, Energy production in anaerobic organisms. *Angew. Chem. Int. Ed. Engl.* **9**, 138–158 (1970).

22. M. A. Pasek, Thermodynamics of prebiotic phosphorylation. *Chem. Rev.* **120**, 4690–4706 (2019).

23. M. Gull, T. Feng, H. A. Cruz, R. Krishnamurthy, M. A. Pasek, Prebiotic chemistry of phosphite: Mild thermal routes to form condensed-P energy currency molecules leading up to the formation of organophosphorus compounds. *Life* **13**, 920 (2023).

24. J. E. Goldford, H. Hartman, T. F. Smith, D. Segrè, Remnants of an ancient metabolism without phosphate. *Cell* **168**, 1126–1134 (2017).

25. M. Preiner, K. Igarashi, K. B. Muchowska, M. Yu, S. J. Varma, K. Kleinermanns, M. K. Nobu, Y. Kamagata, H. Tüysüz, J. Moran, W. F. Martin, A hydrogen-dependent geochemical analogue of primordial carbon and energy metabolism. *Nat. Ecol. Evol.* **4**, 534–542 (2020).

26. K. B. Muchowska, S. J. Varma, J. Moran, Synthesis and breakdown of universal metabolic precursors promoted by iron. *Nature* **569**, 104–107 (2019).

27. T. Beyazay, C. Ochoa-Hernández, Y. Song, K. S. Belthle, W. F. Martin, H. Tüysüz, Influence of composition of nickel-iron nanoparticles for abiotic CO_2 conversion to early prebiotic organics. *Angew. Chem. Int. Ed. Engl* **62**, e202218189 (2023).

28. H. Kaur, S. A. Rauscher, E. Werner, Y. Song, J. Yi, W. Kazöne, W. F. Martin, H. Tüysüz, J. Moran, A prebiotic Krebs cycle analog generates amino acids with H_2 and NH_3 over nickel. *Chem* **10**, 1528–1540 (2024).

29. M. L. Schlikker, M. Brabender, L. Schwander, C. Garcia Garcia, M. Burmeister, S. Metzger, J. Moran, W. F. Martin, Conversion of pyridoxal to pyridoxamine with NH_3 and H_2 on nickel generates a protometabolic nitrogen shuttle under serpentinizing conditions. *FEBS J.* **292**, 3041–3055 (2024).

30. S. J. Varma, K. B. Muchowska, P. Chatelain, J. Moran, Native iron reduces CO_2 to intermediates and end-products of the acetyl-CoA pathway. *Nat. Ecol. Evol.* **2**, 1019–1024 (2018).

31. J. A. Chamberlain, C. R. McLeod, R. J. Traill, G. R. Lachance, Native metals in the Muskox intrusion. *Can. J. Earth Sci.* **2**, 188–215 (1965).

32. C. J. M. Lawley, D. C. Petts, S. E. Jackson, A. Zagorevski, D. G. Pearson, B. A. Kjarsgaard, D. Savard, V. Tschirhart, Precious metal mobility during serpentinization and breakdown of base metal sulphide. *Lithos* **354–355**, 105278 (2020).

33. A. Kutyrev, V. S. Kamenetsky, A. Kontonikas-Charos, D. P. Savelyev, T. Y. Yakich, I. A. Belousov, E. I. Sandimirova, S. V. Moskaleva, Behavior of platinum-group elements during hydrous metamorphism: Constraints from awaruite (Ni_3Fe) mineralization. *Lithosphere* **2023**, 126 (2023).

34. Z. Mao, J. R. Fleming, O. Mayans, J. Freya, D. Schleheck, B. Schink, N. Müller, AMP-dependent phosphite dehydrogenase, a phosphorylating enzyme in dissimilatory phosphite oxidation. *Proc. Natl. Acad. Sci. U.S.A.* **120**, e2309743120 (2023).

35. B. Schink, M. Friedrich, Phosphite oxidation by sulphate reduction. *Nature* **406**, 37 (2000).

36. J. Abramson, J. Adler, J. Dunger, R. Evans, T. Green, A. Pritzel, O. Ronneberger, L. Willmore, A. J. Ballard, J. Bambrick, S. W. Bodenstein, D. A. Evans, C.-C. Hung, M. O'Neill, D. Reiman, K. Tunyasuvunakool, Z. Wu, A. Žemgulytė, E. Arvaniti, C. Beattie, O. Bertolli, A. Bridgland, A. Cherepanov, M. Congreve, A. I. Cowen-Rivers, A. Cowie, M. Figurnov, F. B. Fuchs, H. Gladman, R. Jain, Y. A. Khan, C. M. R. Low, K. Perlin, A. Potapenko, P. Savy, S. Singh, A. Stecula, A. Thillaisundaram, C. Tong, S. Yakneen, E. D. Zhong, M. Zielinski, A. Žídek, V. Bapst, P. Kohli, M. Jaderberg, D. Hassabis, J. M. Jumper, Accurate structure prediction of biomolecular interactions with AlphaFold 3. *Nature* **630**, 493–500 (2024).

37. R. E. Eakin, An approach to the evolution of metabolism. *Proc. Natl. Acad. Sci. U.S.A.* **49**, 360–366 (1963).

38. J. A. Baross, S. E. Hoffman, Submarine hydrothermal vents and associated gradient environments as sites for the origin and evolution of life. *Orig. Life Evol. Biosph.* **15**, 327–345 (1985).

39. W. Martin, M. J. Russell, On the origin of biochemistry at an alkaline hydrothermal vent. *Philos. Trans. R Soc. Lond. B Biol. Sci.* **362**, 1887–1926 (2007).

40. J. C. Fontecilla-Camps, Geochemical continuity and catalyst/cofactor replacement in the emergence and evolution of life. *Angew. Chem. Int. Ed. Engl* **58**, 42–48 (2019).

41. G. E. Fox, Origin and evolution of the ribosome. *Cold Spring Harb. Perspect. Biol.* **2**, a003483 (2010).

42. J. C. Bowman, A. S. Petrov, M. Frenkel-Pinter, P. I. Penev, L. D. Williams, Root of the tree: The significance, evolution, and origins of the ribosome. *Chem. Rev.* **120**, 4848–4878 (2020).

43. D. Huson, J. C. Xavier, M. Steel, Self-generating autocatalytic networks: Structural results, algorithms, and their relevance to early biochemistry. *J. R. Soc. Interface* **21**, 20230732 (2024).

44. B. H. Patel, C. Percivalle, D. J. Ritson, C. D. Duffy, J. D. Sutherland, Common origins of RNA, protein and lipid precursors in a cyanosulfidic protometabolism. *Nat. Chem.* **7**, 301–307 (2015).

45. J. Fairchild, S. Islam, J. Singh, D.-K. Bučar, M. W. Powner, Prebiotically plausible chemoselective pantetheine synthesis in water. *Science* **383**, 911–918 (2024).

46. G. Fuchs, Alternative pathways of carbon dioxide fixation: Insights into the early evolution of life? *Annu. Rev. Microbiol.* **65**, 631–658 (2011).

47. W. F. Martin, Older than genes: The acetyl CoA pathway and origins. *Front. Microbiol.* **11**, 817 (2020).

48. R. Wolfenden, Benchmark reaction rates, the stability of biological molecules in water, and the evolution of catalytic power in enzymes. *Annu. Rev. Biochem.* **80**, 645–667 (2011).

49. A. J. Kluyver, H. J. L. Donker, Die Einheit in der Biochemie. *Chem. Zelle Gewebe* **13**, 134–190 (1926).

50. A. Biester, D. A. Grahame, C. L. Drennan, Capturing a methanogenic carbon monoxide dehydrogenase/acetyl-CoA synthase complex via cryogenic electron microscopy. *Proc. Natl. Acad. Sci. U.S.A.* **121**, e2410995121 (2024).

51. M. D. Yin, O. N. Lemaire, J. G. Rosas Jiménez, M. Belhamri, A. Shevchenko, G. Hummer, T. Wagner, B. J. Murphy, Conformational dynamics of a multienzyme complex in anaerobic carbon fixation. *Science* **387**, 498–504 (2025).

52. T. Wagner, U. Ermler, S. Shima, The methanogenic CO_2 reducing-and-fixing enzyme is bifunctional and contains 46 [4Fe-4S] clusters. *Science* **354**, 114–117 (2016).

53. M. C. Schölmerich, V. Müller, Energy conservation by a hydrogenase-dependent chemiosmotic mechanism in an ancient metabolic pathway. *Proc. Natl. Acad. Sci. U.S.A.* **116**, 6329–6334 (2019).

54. R. K. Thauer, A.-K. Kaster, H. Seedorf, W. Buckel, R. Hedderich, Methanogenic archaea: Ecologically relevant differences in energy conservation. *Nat. Rev. Microbiol.* **6**, 579–591 (2008).

55. F. L. Sousa, W. F. Martin, Biochemical fossils of the ancient transition from geoenergetics to bioenergetics in prokaryotic one carbon compound metabolism. *Biochim. Biophys. Acta Bioenerg.* **1837**, 964–981 (2014).

56. L. D. Modjewski, V. Karavaeva, N. Mrnjavac, M. Knopp, W. F. Martin, F. L. Sousa, Evidence for corrin biosynthesis in the last universal common ancestor. *FEBS J.* **292**, 827–850 (2025).

57. T. M. McCollom, F. Klein, M. Robbins, B. Moskowitz, T. S. Berquó, N. Jöns, W. Bach, A. Templeton, Temperature trends for reaction rates, hydrogen generation, and partitioning of iron during experimental serpentinization of olivine. *Geochim. Cosmochim. Acta* **181**, 175–200 (2016).

58. D. R. Colman, A. S. Templeton, J. R. Spear, E. S. Boyd, Microbial ecology of serpentinite-hosted ecosystems. *ISME J.* **19**, wraf029 (2025).

59. S. Q. Lang, W. J. Brazelton, Habitability of the marine serpentinite subsurface: A case study of the Lost City hydrothermal field. *Philos. Trans. A Math. Phys. Eng. Sci.* **378**, 20180429 (2020).

60. R. J. Mayer, H. Kaur, S. A. Rauscher, J. Moran, Mechanistic insight into metal ion-catalyzed transamination. *J. Am. Chem. Soc.* **143**, 19099–19111 (2021).

61. M. Makarov, A. C. Sanchez Rocha, R. Krystufek, I. Cherepashuk, V. Dzmitruk, T. Charnavets, A. M. Faustino, M. Lebl, K. Fujishima, S. D. Fried, K. Hlouchova, Early selection of the amino acid alphabet was adaptively shaped by biophysical constraints of foldability. *J. Am. Chem. Soc.* **145**, 5320–5329 (2023).

62. U. C. Dave, R. K. Kadeppagari, Alanine dehydrogenase and its applications – a review. *Crit. Rev. Biotechnol.* **39**, 648–664 (2019).

63. R. H. White, L-aspartate semialdehyde and a 6-deoxy-5-ketohexose 1-phosphate are the precursors to the aromatic amino acids in *Methanocaldococcus jannaschii*. *Biochemistry* **43**, 7618–7627 (2004).

64. V. de Crécy-Lagard, G. Phillips, L. L. Grochowski, B. El Yacoubi, F. Jenney, M. W. W. Adams, A. G. Murzin, R. H. White, Comparative genomics guided discovery of two missing archaeal enzyme families involved in the biosynthesis of the pterin moiety of tetrahydromethanopterin and tetrahydrofolate. *ACS Chem. Biol.* **7**, 1807–1816 (2012).

65. T. Rowan, H. C. S. Wood, The biosynthesis of pteridines. Part V. The synthesis of riboflavin from pteridine precursors. *J. Chem. Soc. C* **1968**, 452–458 (1968).

66. R. J. Mayer, J. Moran, Metal ions turn on a stereoselective nonenzymatic reduction of keto acids by the coenzyme NADH. *Chem* **10**, 2564–2576 (2024).

67. C. Degani, M. Halmann, Chemical evolution of carbohydrate metabolism. *Nature* **216**, 1207–1207 (1967).

68. M. A. Keller, A. V. Turchyn, M. Ralser, Non-enzymatic glycolysis and pentose phosphate pathway-like reactions in a plausible Archean ocean. *Mol. Syst. Biol.* **10**, 725 (2014).

69. G. Etiope, M. Schoell, Abiotic gas: Atypical, but not rare. *Elements* **10**, 291–296 (2014).

70. B. Ménez, C. Pisapia, M. Andreani, F. Jamme, Q. P. Vanbellingen, A. Brunelle, L. Richard, P. Dumas, M. Réfrégiers, Abiotic synthesis of amino acids in the recesses of the oceanic lithosphere. *Nature* **564**, 59–63 (2018).

71. M. K. Nobu, R. Nakai, S. Tamazawa, H. Mori, A. Toyoda, A. Ijiri, S. Suzuki, K. Kurokawa, Y. Kamagata, H. Tamaki, Unique H_2-utilizing lithotrophy in serpentinite-hosted systems. *ISME J.* **17**, 95–104 (2023).

72. E. M. Fones, D. R. Colman, E. A. Kraus, R. Stepanauskas, A. S. Templeton, J. R. Spear, E. S. Boyd, Diversification of methanogens into hyperalkaline serpentinizing environments through adaptations to minimize oxidant limitation. *ISME J.* **15**, 1121–1135 (2021).

73. W. Chen, J. Zimmermann, J. Dechent, J. Moran, Nonenzymatic carboxylate phosphorylation in water. *Eur. J. Org. Chem.* **2025**, e202500179 (2025).

74. A. Whicher, E. Camprubi, S. Pinna, B. Herschy, N. Lane, Acetyl phosphate as a primordial energy currency at the origin of life. *Orig. Life Evol. Biosph.* **48**, 159–179 (2018).

75. E. Frouin, A. Lecoeuvre, F. Armougom, M. O. Schrenk, G. Erauso, Comparative metagenomics highlight a widespread pathway involved in catabolism of phosphonates in marine and terrestrial serpentinizing ecosystems. *mSystems* **7**, e00328–e00322 (2022).

76. J. S. Boden, S. M. Som, W. J. Brazelton, R. E. Anderson, E. E. Stüeken, Evaluating serpentinization as a source of phosphite to microbial communities in hydrothermal vents. *Geobiology* **23**, e70016 (2025).

77. B. D. Bennett, E. H. Kimball, M. Gao, R. Osterhout, S. J. Van Dien, J. D. Rabinowitz, Absolute metabolite concentrations and implied enzyme active site occupancy in *Escherichia coli*. *Nat. Chem. Biol.* **5**, 593–599 (2009).

78. M. L. Schlikker, N. K. Hoffmann, W. F. Martin, Hydrothermal origin of metabolic phosphorylation. bioRxiv 2025.12.19.695421 [Preprint] (2025) [cited 2025]. https://doi.org/10.64898/2025.12.19.695421.

79. F. Klein, V. Le Roux, Quantifying the volume increase and chemical exchange during serpentinization. *Geology* **48**, 552–556 (2020).

80. H. M. Lamadrid, J. D. Rimstidt, E. M. Schwarzenbach, F. Klein, S. Ulrich, A. Dolocan, R. J. Bodnar, Effect of water activity on rates of serpentinization of olivine. *Nat. Commun.* **8**, 16107 (2017).

81. R. Tamblyn, J. Hermann, Geological evidence for high H_2 production from komatiites in the Archaean. *Nat. Geosci.* **16**, 1194–1199 (2023).

82. D. P. Henriques Pereira, J. Leethaus, T. Beyazay, A. do Nascimento Vieira, K. Kleinermanns, H. Tüysüz, W. F. Martin, M. Preiner, Role of geochemical protoenzymes (geozymes) in primordial metabolism: Specific abiotic hydride transfer by metals to the biological redox cofactor NAD^+. *FEBS J.* **289**, 3148–3162 (2022).

83. K. Yang, W. W. Metcalf, A new activity for an old enzyme: *Escherichia coli* bacterial alkaline phosphatase is a phosphite-dependent hydrogenase. *Proc. Natl. Acad. Sci. U.S.A.* **101**, 7919–7924 (2004).

84. J. F. Hartwig, *Organotransition Metal Chemistry* (MIT Press, 2010).

85. R. H. White, Biosynthesis of the methanogenic cofactors. *Vitam. Horm.* **61**, 299–337 (2001).

86. A. Kirschning, On the evolution of coenzyme biosynthesis. *Nat. Prod. Rep.* **39**, 2175–2191 (2022).

87. W. Buckel, R. K. Thauer, Flavin-based electron bifurcation, a new mechanism of biological energy coupling. *Chem. Rev.* **118**, 3862–3886 (2018).

88. E. S. Boyd, M. J. Amenabar, S. Poudel, A. S. Templeton, Bioenergetic constraints on the origin of autotrophic metabolism. *Philos. Trans. A Math. Phys. Eng. Sci.* **378**, 20190151 (2020).

89. M. Brabender, D. P. Henriques Pereira, N. Mrnjavac, M. L. Schlikker, Z.-I. Kimura, J. Sucharitakul, K. Kleinermanns, H. Tüysüz, W. Buckel, M. Preiner, W. F. Martin, Ferredoxin

reduction by hydrogen with iron functions as an evolutionary precursor of flavin-based electron bifurcation. *Proc. Natl. Acad. Sci. U.S.A.* **121**, e2318969121 (2024).

90. S. W. Ragsdale, Pyruvate ferredoxin oxidoreductase and its radical intermediate. *Chem. Rev.* **103**, 2333–2346 (2003).

91. T. Svetlitchnaia, V. Svetlitchnyi, O. Meyer, H. Dobbek, Structural insights into methyltransfer reactions of a corrinoid iron–sulfur protein involved in acetyl-CoA synthesis. *Proc. Natl. Acad. Sci. U.S.A.* **103**, 14331–14336 (2006).

92. S. K. Rout, S. Wunnava, M. Krepl, G. Cassone, J. E. Šponer, C. B. Mast, M. W. Powner, D. Braun, Amino acids catalyse RNA formation under ambient alkaline conditions. *Nat. Commun.* **16**, 5193 (2025).

93. J.-H. Jeoung, H. Dobbek, Structural basis of cyanide inhibition of Ni,Fe-containing carbon monoxide dehydrogenase. *J. Am. Chem. Soc.* **131**, 9922–9923 (2009).

94. S. A. Benner, A. D. Ellington, A. Tauer, Modern metabolism as a palimpsest of the RNA world. *Proc. Natl. Acad. Sci. U.S.A.* **86**, 7054–7058 (1989).

95. M. Piejko, J. E. Alfonso-Ramos, J. Moran, T. Stuyver, Abiotic ribonucleoside formation in aqueous microdroplets: Mechanistic exploration, acidity, and electric field effects. *Chem. A Eur. J.* **3**, e202400093 (2025).

96. M. Rother, A. Resch, R. Wilting, A. Böck, Selenoprotein synthesis in archaea. *Biofactors* **14**, 75–83 (2001).

97. K. Evans, S. M. Reddy, R. Merle, D. Fougerouse, W. D. A. Rickard, D. W. Saxey, J.-W. Park, L. Doucet, F. Jourdan, The origin of platinum group minerals in oceanic crust. *Geology* **51**, 554–558 (2023).

98. H. Atomi, H. Tomita, T. Ishibashi, Y. Yokooji, T. Imanaka, CoA biosynthesis in archaea. *Biochem. Soc. Trans.* **41**, 427–431 (2013).

99. R. A. Jensen, Enzyme recruitment in evolution of new function. *Annu. Rev. Microbiol.* **30**, 409–425 (1976).

100. H. Adkins, H. I. Cramer, The use of nickel as a catalyst for hydrogenation. *J. Am. Chem. Soc.* **52**, 4349–4358 (1930).

101. F. P. Rosenbaum, V. Müller, Energy conservation under extreme energy limitation: The role of cytochromes and quinones in acetogenic bacteria. *Extremophiles* **25**, 413–424 (2021).

102. C. Bräsen, D. Esser, B. Rauch, B. Siebers, Carbohydrate metabolism in archaea: Current insights into unusual enzymes and pathways and their regulation. *Microbiol. Mol. Biol. Rev.* **78**, 89–175 (2014).

103. J. Brueckner, W. F. Martin, Bacterial genes outnumber archaeal genes in eukaryotic genomes. *Genome Biol. Evol.* **12**, 282–292 (2020).

104. B. Buchfink, C. Xie, D. H. Huson, Fast and sensitive protein alignment using DIAMOND. *Nat. Methods* **12**, 59–60 (2015).

105. The UniProt Consortium, UniProt: The universal protein knowledgebase in 2025. *Nucleic Acids Res.* **53**, D609–D617 (2025).

106. J. Jumper, R. Evans, A. Pritzel, T. Green, M. Figurnov, O. Ronneberger, K. Tunyasuvunakool, R. Bates, A. Žídek, A. Potapenko, A. Bridgland, C. Meyer, S. A. A. Kohl, A. J. Ballard, A. Cowie, B. Romera-Paredes, S. Nikolov, R. Jain, J. Adler, T. Back, S. Petersen, D. Reiman, E. Clancy, M. Zielinski, M. Steinegger, M. Pacholska, T. Berghammer, S. Bodenstein, D. Silver, O. Vinyals, A. W. Senior, K. Kavukcuoglu, P. Kohli, D. Hassabis, Highly accurate protein structure prediction with AlphaFold. *Nature* **596**, 583–589 (2021).

107. M. Mirdita, K. Schütze, Y. Moriwaki, L. Heo, S. Ovchinnikov, M. Steinegger, ColabFold: Making protein folding accessible to all. *Nat. Methods* **19**, 679–682 (2022).

108. M. Varadi, D. Bertoni, P. Magana, U. Paramval, I. Pidruchna, M. Radhakrishnan, M. Tsenkov, S. Nair, M. Mirdita, J. Yeo, O. Kovalevskiy, K. Tunyasuvunakool, A. Laydon, A. Žídek, H. Tomlinson, D. Hariharan, J. Abrahamson, T. Green, J. Jumper, E. Birney, M.

Steinegger, D. Hassabis, S. Velankar, AlphaFold Protein Structure Database in 2024: Providing structure coverage for over 214 million protein sequences. *Nucleic Acids Res.* **52**, D368–D375 (2024).

109. C. Zhang, M. Shine, A. M. Pyle, Y. Zhang, US-align: Universal structure alignments of proteins, nucleic acids, and macromolecular complexes. *Nat. Methods* **19**, 1109–1115 (2022).

110. J. Xu, Y. Zhang, How significant is a protein structure similarity with TM-score = 0.5? *Bioinformatics* **26**, 889–895 (2010).

111. D. T. Gallagher, H. G. Monbouquette, I. Schröder, H. Robinson, M. J. Holden, N. N. Smith, Structure of alanine dehydrogenase from *Archaeoglobus*: Active site analysis and relation to bacterial cyclodeaminases and mammalian μ crystallin. *J. Mol. Biol.* **342**, 119–130 (2004).

112. Y. Darzi, I. Letunic, P. Bork, T. Yamada, iPath3.0: Interactive pathways explorer v3. *Nucleic Acids Res.* **46**, W510–W513 (2018).

113. N. Yutin, P. Puigbò, E. V. Koonin, Y. I. Wolf, Phylogenomics of prokaryotic ribosomal proteins. *PLOS ONE* **7**, e36972 (2012).

114. N. Ban, R. Beckmann, J. H. D. Cate, J. D. Dinman, F. Dragon, S. R. Ellis, D. L. J. Lafontaine, L. Lindahl, A. Liljas, J. M. Lipton, M. A. McAlear, P. B. Moore, H. F. Noller, J. Ortega, V. G. Panse, V. Ramakrishnan, C. M. T. Spahn, T. A. Steitz, M. Tchorzewski, D. Tollervey, M. Yusupov, A new system for naming ribosomal proteins. *Curr. Opin. Struct. Biol.* **24**, 165–169 (2014).

115. M. Y. Galperin, Y. I. Wolf, S. K. Garushyants, R. Vera Alvarez, E. V. Koonin, Non-essential ribosomal proteins in bacteria and archaea identified using COGs. *J. Bacteriol.* **203**, e00058–e00021 (2021).

116. A. Sas-Chen, J. M. Thomas, D. Matzov, M. Taoka, K. D. Nance, R. Nir, K. M. Bryson, R. Shachar, G. L. S. Liman, B. W. Burkhart, S. Thalalla Gamage, Y. Nobe, C. A. Briney, M. J. Levy, R. T. Fuchs, G. B. Robb, J. Hartmann, S. Sharma, Q. Lin, L. Florens, M. P. Washburn,

T. Isobe, T. J. Santangelo, M. Shalev-Benami, J. L. Meier, S. Schwartz, Dynamic RNA acetylation revealed by quantitative cross-evolutionary mapping. *Nature* **583**, 638–643 (2020).

117. Z. L. Watson, F. R. Ward, R. Méheust, O. Ad, A. Schepartz, J. F. Banfield, J. H. D. Cate, Structure of the bacterial ribosome at 2 Å resolution. *eLife* **9**, e60482 (2020).

118. D. H. Huson, J. C. Xavier, M. A. Steel, CatReNet: Interactive analysis of (auto-)catalytic reaction networks. *Bioinformatics* **40**, btae515 (2024).

119. M. Steel, W. Hordijk, J. Smith, Minimal autocatalytic networks. *J. Theor. Biol.* **332**, 96–107 (2013).

120. P. Shannon, A. Markiel, O. Ozier, N. S. Baliga, J. T. Wang, D. Ramage, N. Amin, B. Schwikowski, T. Ideker, Cytoscape: A software environment for integrated models of biomolecular interaction networks. *Genome Res.* **13**, 2498–2504 (2003).

121. B. Hippler, R. K. Thauer, The energy conserving methyltetrahydromethanopterin: Coenzyme M methyltransferase complex from methanogenic archaea: Function of the subunit MtrH. *FEBS Lett.* **449**, 165–168 (1999).

122. E. Biegel, S. Schmidt, V. Müller, Genetic, immunological and biochemical evidence for a Rnf complex in the acetogen *Acetobacterium woodii*. *Environ. Microbiol.* **11**, 1438–1443 (2009).

123. F. Mayer, V. Müller, Adaptations of anaerobic archaea to life under extreme energy limitation. *FEMS Microbiol. Rev.* **38**, 449–472 (2014).

124. M. Sobti, J. L. Walshe, D. Wu, R. Ishmukhametov, Y. C. Zeng, C. V. Robinson, R. M. Berry, A. G. Stewart, Cryo-EM structures provide insight into how *E. coli* F1Fo ATP synthase accommodates symmetry mismatch. *Nat. Commun.* **11**, 2615 (2020).

125. L. Zhou, L. A. Sazanov, Structure and conformational plasticity of the intact *Thermus thermophilus* V/A-type ATPase. *Science* **365**, eaaw9144 (2019).

126. C. Huguet, S. Fietz, A. Rosell-Melé, X. Daura, L. Costenaro, Molecular dynamics simulation study of the effect of glycerol dialkyl glycerol tetraether hydroxylation on membrane thermostability. *Biochim. Biophys. Acta Biomembr.* **1859**, 966–974 (2017).

127. S. Vitt, S. Prinz, M. Eisinger, U. Ermler, W. Buckel, Purification and structural characterization of the Na^+–translocating ferredoxin:NAD^+ reductase (Rnf) complex of *Clostridium tetanomorphum*. *Nat. Commun.* **13**, 6315 (2022).

128. I. Aziz, K. Kayastha, S. Kaltwasser, J. Vonck, S. Welsch, B. J. Murphy, J. Kahnt, D. Wu, T. Wagner, S. Shima, U. Ermler, Structural and mechanistic basis of the central energy-converting methyltransferase complex of methanogenesis. *Proc. Natl. Acad. Sci. U.S.A.* **121**, e2315568121 (2024).

129. T. Wagner, U. Ermler, S. Shima, MtrA of the sodium ion pumping methyltransferase binds cobalamin in a unique mode. *Sci. Rep.* **6**, 28226 (2016).

130. D. P. Tieleman, H. J. C. Berendsen, A molecular dynamics study of the pores formed by *Escherichia coli* OmpF porin in a fully hydrated palmitoyloleoylphosphatidylcholine bilayer. *Biophys. J.* **74**, 2786–2801 (1998).

131. W. F. Martin, K. Kleinermanns, *The Geochemical Origin of Microbes* (CRC Press, 2024).

132. J. Krissansen-Totton, G. N. Arney, D. C. Catling, Constraining the climate and ocean pH of the early Earth with a geological carbon cycle model. *Proc. Natl. Acad. Sci. U.S.A.* **115**, 4105–4110 (2018).

133. D. He, X. Wang, Y. Yang, R. He, H. Zhong, Y. Wang, B. Han, F. Jin, Hydrothermal synthesis of long-chain hydrocarbons up to C24 with $NaHCO_3$-assisted stabilizing cobalt. *Proc. Natl. Acad. Sci. U.S.A.* **118**, e2115059118 (2021).

134. P. Zhu, C. Wang, J. Lang, D. He, F. Jin, Prebiotic synthesis of microdroplets from formate over a bimetallic cobalt–nickel nanomotif. *J. Am. Chem. Soc.* **146**, 25005–25015 (2024).

135. F. Yu, J. Fei, Y. Jia, T. Wang, W. F. Martin, J. Li, Chemiosmotic ATP synthesis by minimal protocells. *Cell Rep. Phys. Sci.* **6**, 102461 (2025).

136. K. Schlegel, C. Welte, U. Deppenmeier, V. Müller, Electron transport during aceticlastic methanogenesis by *Methanosarcina acetivorans* involves a sodium-translocating Rnf complex. *FEBS J.* **279**, 4444–4452 (2012).

137. Y. Nagaosa, E. Aoyama, Catalytic oxidation of phosphite and hypophosphite to phosphate on Pd/activated carbon powder. *Carbon* **39**, 2087–2088 (2001).

138. A. Kirschning, Why pyridoxal phosphate could be a functional predecessor of thiamine pyrophosphate and speculations on a primordial metabolism. *RSC Chem. Biol.* **5**, 508–517 (2024).

139. K. S. Belthle, W. F. Martin, H. Tüysüz, Synergistic effects of silica-supported iron–cobalt catalysts for CO_2 reduction to prebiotic organics. *ChemCatChem* **16**, e202301218 (2024).

140. J. Nan, S. Luo, Q. P. Tran, A. C. Fahrenbach, W.-N. Lu, Y. Hu, Z. Yin, J. Ye, M. J. Van Kranendonk, Iron sulfide-catalyzed gaseous CO_2 reduction and prebiotic carbon fixation in terrestrial hot springs. *Nat. Commun.* **15**, 10280 (2024).

141. E. P. Reeves, J. S. Seewald, Hydrothermal carbon reduction in the absence of minerals. *Geochim. Cosmochim. Acta* **381**, 60–74 (2024).

142. Y. Song, H. Tüysüz, CO_2 fixation to prebiotic intermediates over heterogeneous catalysts. *Acc. Chem. Res.* **57**, 2038–2047 (2024).

143. R. Hudson, R. de Graaf, M. Strandoo Rodin, A. Ohno, N. Lane, S. E. McGlynn, Y. M. A. Yamada, R. Nakamura, L. M. Barge, D. Braun, V. Sojo, CO_2 reduction driven by a pH gradient. *Proc. Natl. Acad. Sci. U.S.A.* **117**, 22873–22879 (2020).

144. Q. Dherbassy, R. J. Mayer, K. B. Muchowska, J. Moran, Metal-pyridoxal cooperativity in nonenzymatic transamination. *J. Am. Chem. Soc.* **145**, 13357–13370 (2023).

145. D. E. Metzler, E. E. Snell, Some transamination reactions involving vitamin B6. *J. Am. Chem. Soc.* **74**, 979–983 (1952).

146. T. Nakajima, Y. Yabushita, I. Tabushi, Amino acid synthesis through biogenetic-type CO_2 fixation. *Nature* **256**, 60–61 (1975).

147. S. A. Sanden, R. Yi, M. Hara, S. E. McGlynn, Simultaneous synthesis of thioesters and iron–sulfur clusters in water: Two universal components of energy metabolism. *Chem. Commun.* **56**, 11989–11992 (2020).

148. T. Beyazay, K. S. Belthle, C. Farès, M. Preiner, J. Moran, W. F. Martin, H. Tüysüz, Ambient temperature CO_2 fixation to pyruvate and subsequently to citramalate over iron and nickel nanoparticles. *Nat. Commun.* **14**, 570 (2023).

149. Y. Song, T. Beyazay, H. Tüysüz, Effect of alkali- and alkaline-earth-metal promoters on silica-supported Co–Fe alloy for autocatalytic CO_2 fixation. *Angew. Chem. Int. Ed. Engl.* **63**, e202316110 (2024).

150. K. B. Muchowska, S. J. Varma, E. Chevallot-Beroux, L. Lethuillier-Karl, G. Li, J. Moran, Metals promote sequences of the reverse Krebs cycle. *Nat. Ecol. Evol.* **1**, 1716–1721 (2017).

151. O. Yañez, R. Cabrera, R. Pino-Rios, C. Sepúlveda, X. A. López-Cortés, F. D. González-Nilo, M. C. Ravanal, Metal-catalyzed decarboxylation of oxaloacetic acid studied in silico and in vitro: Implications for enzymatic and prebiotic catalysis. *J. Mol. Struct.* **1326**, 141105 (2025).

152. Z. Tang, X. Liu, Y. Yang, F. Jin, Recent advances in CO_2 reduction with renewable reductants under hydrothermal conditions: Towards efficient and net carbon benefit CO_2 conversion. *Chem. Sci.* **15**, 9927–9948 (2024).

153. C. He, G. Tian, Z. Liu, S. Feng, A mild hydrothermal route to fix carbon dioxide to simple carboxylic acids. *Org. Lett.* **12**, 649–651 (2010).

154. Z. Liu, G. Tian, S. Zhu, C. He, H. Yue, S. Feng, Ready hydrothermal reactions from carbon dioxide to methane. *ACS Sustain. Chem. Eng.* **1**, 313–315 (2013).
